# Polymerase independent repression of *FoxO1* transcription by sequence-specific PARP1 binding to *FoxO1* promoter

**DOI:** 10.1038/s41419-020-2265-y

**Published:** 2020-01-28

**Authors:** Yu-Nan Tian, Hua-Dong Chen, Chang-Qing Tian, Ying-Qing Wang, Ze-Hong Miao

**Affiliations:** 1 Division of Anti-Tumor Pharmacology, State Key Laboratory of Drug Research, Shanghai Institute of Materia Medica, Chinese Academy of Sciences, Shanghai, 201203 China; 2 University of Chinese Academy of Sciences, No.19A Yuquan Road, Beijing, 100049 China; 3 Open Studio for Druggability Research of Marine Natural Products, Pilot National Laboratory for Marine Science and Technology, Qingdao, Shandong 266237 China

**Keywords:** Oncogenes, Transcriptional regulatory elements

## Abstract

Poly(ADP-ribose) polymerase 1 (PARP1) regulates gene transcription in addition to functioning as a DNA repair factor. Forkhead box O1 (FoxO1) is a transcription factor involved in extensive biological processes. Here, we report that PARP1 binds to two separate motifs on the *FoxO1* promoter and represses its transcription in a polymerase-independent manner. Using *PARP1*-knock out (KO) cells, wild-type-*PARP1*-complemented cells and catalytic mutant *PARP1*^*E988K*^-reconstituted cells, we investigated transcriptional regulation by PARP1. *PARP1* loss led to reduced DNA damage response and ~362-fold resistance to five PARP inhibitors (PARPis) in Ewing sarcoma cells. RNA sequencing showed 492 differentially expressed genes in a *PARP1*-KO subline, in which the *FoxO1* mRNA levels increased up to more than five times. The change in the FoxO1 expression was confirmed at both mRNA and protein levels in different *PARP1*-KO and complemented cells. Moreover, exogenous *PARP1* overexpression reduced the endogenous FoxO1 protein in RD-ES cells. Competitive EMSA and ChIP assays revealed that PARP1 specifically bound to the *FoxO1* promoter. DNase I footprinting, mutation analyses, and DNA pulldown FREP assays showed that PARP1 bound to two particular nucleotide sequences separately located at −813 to −826 bp and −1805 to −1828 bp regions on the *FoxO1* promoter. Either the PARPi olaparib or the *PARP1* catalytic mutation (E988K) did not impair the repression of PARP1 on the *FoxO1* expression. Exogenous *FoxO1* overexpression did not impair cellular PARPi sensitivity. These findings demonstrate a new PARP1-gene promoter binding mode and a new transcriptional *FoxO1* gene repressor.

## Introduction

Poly(ADP-ribose) polymerase 1 (PARP1) plays critical roles in DNA repair *via* catalyzing the transfer of the ADP-ribosyl group of NAD^+^ onto acceptor proteins (including PARP1 itself) to form poly(ADP-ribose) polymers, a process known as poly(ADP-ribosyl)ation (PARylation)^1–3^. PARP1 inhibitors (PARPis) have been shown to selectively kill homologous recombination repair (HRR) deficient cancer cells^1–3^ by increasing PARP1-DNA binding due to suppression of autoPARylation of PARP1 on DNA^4^. Four PARPis (olaparib, rucaparib, niraparib, and talazoparib) have been clinically used for cancer therapy, and more are undergoing clinical or preclinical tests^3,5–11^. Our recent studies have revealed that treatments of cancer cells with PARPis reduce the expression of 53BP1 or enhance the expression of COX-2, BIRC3, and SAMHD1, which contributes to cellular drug resistance^12,13^. These findings suggest that transcriptional regulation by PARP1 appears to affect the cellular sensitivity to PARPis or other anticancer drugs. Down-regulation of *BRCA2* expression by PARP1 in an enzymatic activity dependent manner^14^ provides an additional supporting clue for this.

PARP1 has been reported to regulate gene transcription in several ways^15–18^. The transcriptional regulation by PARP1 is dependent on or independent of its polymerase activity and varies in gene-, cell type-, and context-specific manners^15,16^. All these indicate that the PARP1-mediated transcriptional regulation is complicated and unpredictable based on present knowledge. Therefore, further investigations, such as its DNA sequence dependency and its correlations with cellular PARPi sensitivity, are required.

We previously established *PARP1*-KO sublines of Ewing sarcoma RD-ES and SK-ES-1 cell lines, which were denoted as RD/KO1, RD/KO2, SK/KO1, and SK/KO2 separately^4^. Here, we first characterized these sublines about their responses to PARPis. Then, we conducted RNA profiling in both RD-ES and RD/KO1 cells to find changes in mRNA levels due to *PARP1* loss. Following a series of analyses and verifications, Forkhead box O1 (*FoxO1*) was selected for further explorations because the *PARP1* KO significantly increases its mRNA and protein levels in different cell lines, which was partly reversed by *PARP1* complementation. Subsequently, we demonstrated by electrophoretic mobility shift (EMSA) and chromatin immunoprecipitation (ChIP) that PARP1 binds to the *FoxO1* promoter. This binding was further confirmed to be DNA sequence specific by DNase I footprinting assays, EMSA, and flanking restriction enhanced pulldown (FREP) assays. Finally, the transcriptional inhibition of *FoxO1* by PARP1 was shown to be independent of its enzymatic activity and cellular PARPi sensitivity.

## Results

### Characterization of *PARP1*-knockout variants of RD-ES and SK-ES-1 cells

**Fig. 1:**
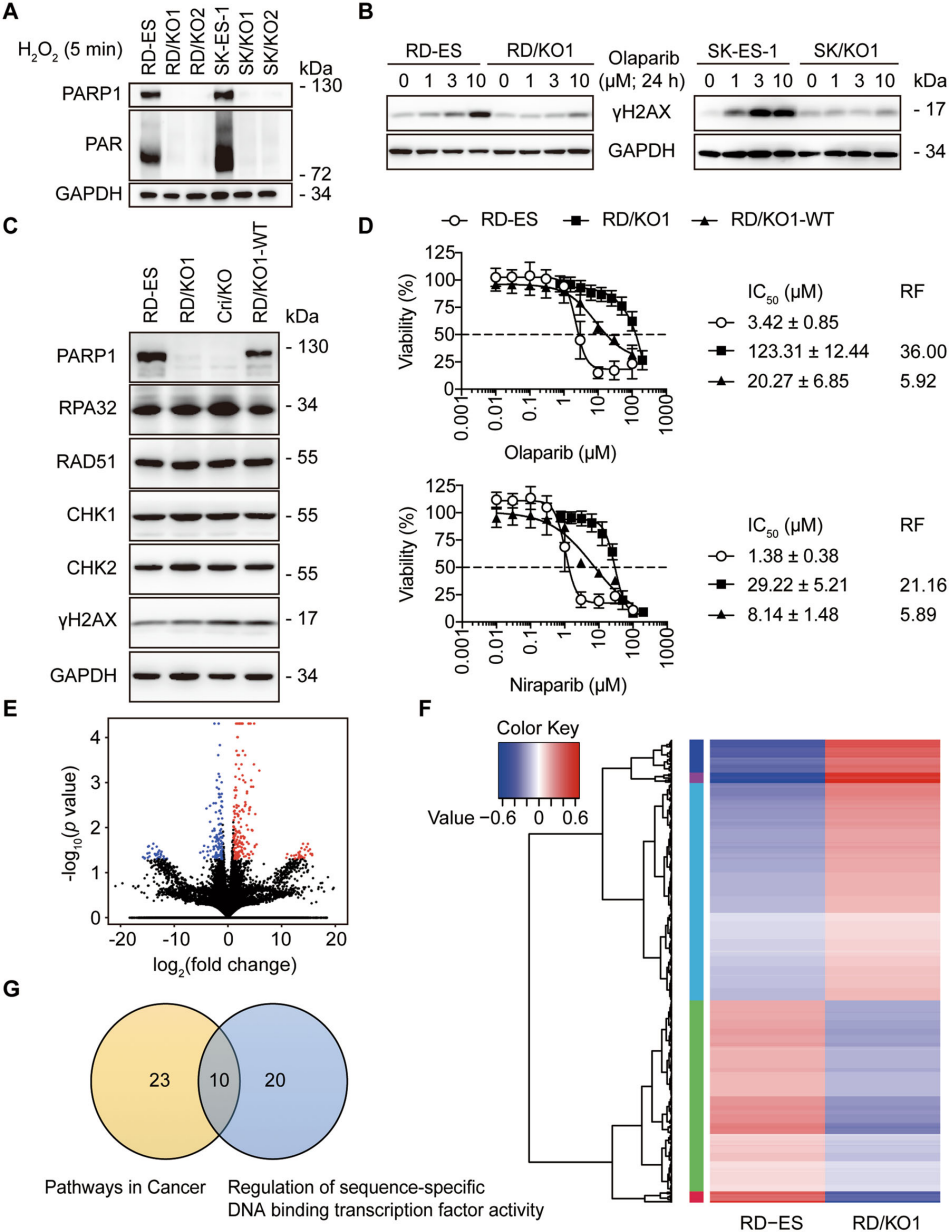
Characterization of *PARP1*-knockout (KO) (*PARP1*/KO) variants. **a** Levels of PARP1 and PAR were detected by western blotting in different *PARP1*/KO variants (KO1 and KO2) of RD-ES and SK-ES-1 cells exposed to 200 μM H_2_O_2_ for 5 min. **b** Accumulation of γH2AX was reduced in *PARP1*/KO cells relative to parental cells treated with olaparib (0, 1, 3, or 10 μM). **c** Levels of DNA repair-related proteins in the RD-ES, RD/KO1, Cri/KO, and RD/KO1-WT cells were determined by western blotting. **d** Changes in PARPi sensitivity in response to *PARP1* loss and *PARP1* reconstitution. IC_50_ values from three independent experiments were expressed as mean ± SD. Error bars represent the SD. The resistance factor (RF) is the ratio of the averaged IC_50_ value of indicated PARPi in given cells to that of the same PARPi in RD-ES cells. **e** Volcano plots of the differentially expressed genes in RD/KO1 cells [log_2_ fold change >1 with statistical significance (*p* < 0.05)] detected by RNA-seq. Significantly upregulated and downregulated genes were colored in red and blue, respectively. X axis: log_2_ fold change of gene expression. Y axis: statistical significance of the differential expression in the scale of −log_10_ (*p* value). **f** Hierarchical clustered heatmap of differentially expressed genes in *PARP1* loss cells: rows represent cell lines and columns represent genes. Genes with similar expression patterns are within the same cluster and close to each other, and they may have similar functions or participate in the same biological processes. In clustering analysis, high expression and low expression genes are colored in red and blue, respectively (Genes were shown in Supplementary Table S1 from top to bottom). **g** Differentially expressed genes in RD/KO1 cells involved in “pathways in cancer” and “regulation of sequence-specific DNA binding transcription factor activity” were plotted in a Venn diagram to display commonly affected genes (Genes were shown in Supplementary Table S2).

Ewing sarcoma is the fifth highest PARP1-expressing malignancy^19^. To investigate the transcriptional regulation by PARP1, we used cellular models generated from Ewing sarcoma RD-ES and SK-ES-1 cells by knocking out the *PARP1* gene, denoted as RD/KO1, RD/KO2, SK/KO1, and SK/KO2^4^. All these clones almost completely lost their PARP1 expression and PAR formation (Fig. 1a) and displayed ~362-fold resistance to five PARPis^4^. The treatment with PARPi olaparib led to apparently less increase in levels of γH2AX [a marker of DNA double-strand breaks (DSB)]^20^ in the *PARP1*-deficient cells than in their respective parental cells (Fig. 1b). The levels of central components involved in DNA damage response (DDR) such as RPA32, RAD51, CHK1, and CHK2 in both RD/KO1 cells and CRISPR-mediated *PARP1* KO (Cri/KO) cells were similar to that of the parent RD-ES cells and the wild-type-*PARP1*-complemented RD/KO1 (RD/KO1-WT) cells^4^ (Fig. 1c). Notably, complementation with WT-*PARP1* only partially restored PARPi sensitivity in RD/KO1-WT cells (Fig. 1d).

### Depletion of *PARP1* increases FoxO1 expression

To identify target genes transcriptionally regulated by PARP1, we conducted transcription profiling in RD/KO1 cells and parental cells by RNA-seq. The results showed that the expression of 492 genes changed significantly in *PARP1*/KO cells [log_2_ fold change >1 with statistical significance (*p* < 0.05)]. The volcano plot (Fig. 1e) and the hierarchical clustered heatmap (Fig. 1f) revealed that among these expression-changed genes, 277 genes were upregulated, and 215 genes were downregulated (These genes in hierarchical clustered heatmap were shown in Supplementary Table S1 from top to bottom.) Among them, KEGG analysis further demonstrated that 23 genes were involved in “pathways in cancer” while the GO analysis indicated that 20 genes participated in “regulation of sequence-specific DNA binding transcription factor activity.” Interestingly, 10 genes were common to both (Fig. 1g). Among the 10 genes, the expression of seven genes changed obviously (log_2_ fold change >2), including TNF alpha induced protein 3 (*TNFAIP3*; up), nuclear factor kappa B subunit 2 (*NF-κB2;* up), nuclear factor kappa B subunit 1 (*NF-κB1*; up), NF-κB inhibitor alpha (*NF-κBIA*, also known as *IκBα*; up), *FoxO1* (up), inhibitor of nuclear factor kappa B kinase subunit beta (*IκBKB*; down), and androgen receptor (*AR*; down) (Supplementary Table S2).

**Fig. 2:**
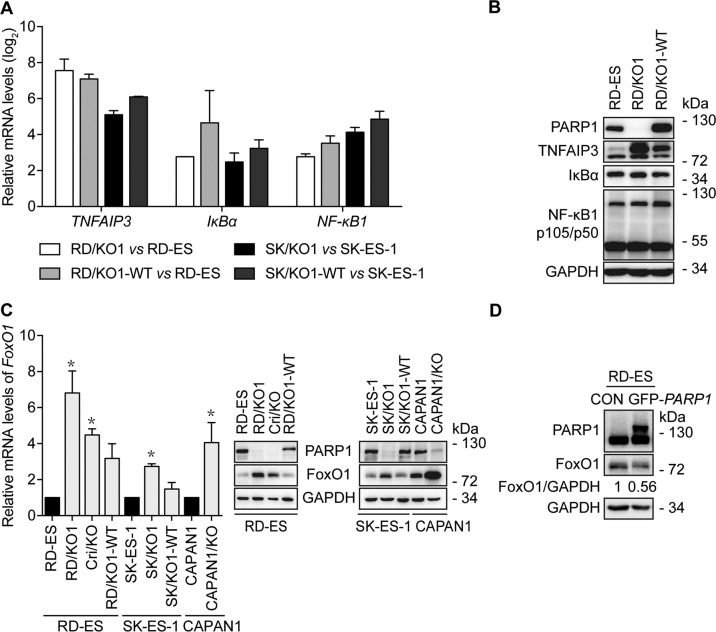
*PARP1* loss increases FoxO1 expression. **a** Confirmation of some results from RNA-seq by RT-qPCR in different cells. Log_2_ mRNA levels of *TNFAIP3*, *IκBα*, and *NF-κB1* in *PARP1*-KO or complemented cells were normalized to that in corresponding parental cells. Error bars represent the SD. **b** Confirmation of some results from RNA-seq by western blotting in indicated cells. **c** Loss of *PARP1* increased mRNA and protein levels of FoxO1, which was prevented, at least partially, by *PARP1* complementation. The mRNA levels of *FoxO1* were detected by RT-qPCR and normalized to that in the corresponding parental cells; Error bars represent the SD. *, *p* < 0.05. Protein levels of FoxO1 were detected by western blotting. **d** After RD-ES cells were transfected with GFP-*PARP1* cDNA for 72 h, protein levels of FoxO1 were determined by western blotting. The relative FoxO1 levels were presented as the ratio of (FoxO1/GAPDH)_GFP-*PARP1*_/(FoxO1/GAPDH)_CON_ when the value of (FoxO1/GAPDH)_CON_ was normalized as 1.

PARP1 has been reported to support the transcriptional function of AR^17^. Additionally, the above results show that genes related to NF-κB1 signaling might be affected most due to *PARP1* loss. Thus, to verify the results from the transcription profiling, we used parental cells (RD-ES and SK-ES-1), *PARP1*-KO cells (RD/KO1 and SK/KO1), and their WT-*PARP1*-complemented cells (RD/KO1-WT and SK/KO1-WT)^4^. RT-qPCR revealed that though the mRNA levels of *TNFAIP3*, *IκBα*, and *NF-κB1* were strikingly elevated due to *PARP1* loss, complementation with WT-*PARP1* did not reduce their elevation (Fig. 2a). Moreover, western blotting further showed that *PARP1* loss caused increased TNFAIP3 protein levels but no change in IκBα or NF-κB1 levels. In addition, *PARP1* reconstitution led to no (IκBα or NF-κB1) or only weak (TNFAIP3) changes at the protein levels (Fig. 2b). These inconsistent results indicated that genes related to NF-κB1 signaling might not be regulated by PARP1, at least not in detected Ewing sarcoma cells.

Therefore, we turned to *FoxO1*, which encodes a transcription factor that regulates gene expression, controlling various cellular processes^21^. For changes in *FoxO1* gene expression caused by either *PARP1* loss or complementation in different cells, both RT-qPCR and western blotting provided consistent results to the transcription profiling (Fig. 2c and Supplementary Table S1). RNA-seq revealed increased *FoxO1* mRNA levels up to more than five times in RD/KO1 cells (Supplementary Table S1), while RT-qPCR showed 2.7–6.8-fold increases in *PARP1*-KO Ewing sarcoma RD/KO1, Cri/KO and SK/KO1 cells and pancreatic CAPAN1/KO cells (Fig. 2c, left). Similar increases in FoxO1 protein levels were observed in these cells (Fig. 2c, middle and right). Notably, *PARP1* complementation could reduce, though not eliminate, the *PARP1* loss-mediated increase in either mRNA or protein levels of this gene (Fig. 2c). To further validate these results, we overexpressed *PARP1* by transfecting GFP-*PARP1* into RD-ES. The result showed that exogenous PARP1 overexpression could reduce endogenous FoxO1 protein levels by 44% (Fig. 2d). These results indicate that PARP1 negatively regulates *FoxO1* gene transcription. Moreover, transcriptional regulation of *FoxO1* by PARP1 is not just limited to Ewing sarcoma cells; similar changes were observed in CAPAN1 cells (Fig. 2c). Furthermore, though *FoxO1* has been reported to be a direct target gene of EWS-FLI1 in Ewing sarcoma cells^21^, this regulation is independent of EWS-FLI1 because it does not exist in CAPAN1 cells.

### PARP1 binding to the *FoxO1* promoter

**Fig. 3:**
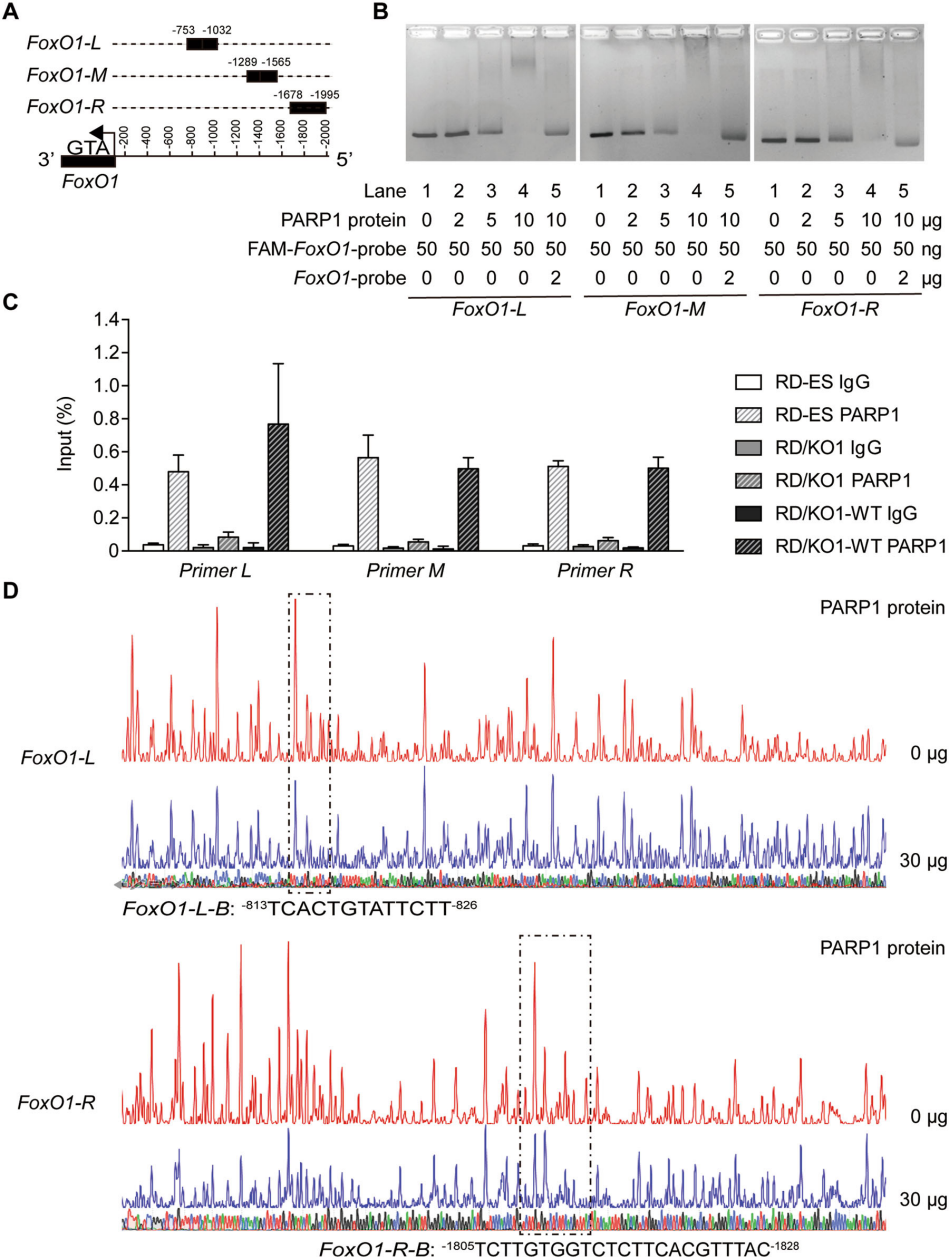
PARP1 binding to particular regions on the *FoxO1* promoter. **a** Schematic representation of the locations of particular nucleotide fragments (*FoxO1-L*, *-M*, and *-R*) on the *FoxO1* promoter analyzed by EMSA. **b** The binding of purified PARP1 to particular nucleotide fragments *(FoxO1-L, -M*, and *-R)* were analyzed by EMSA. For the competition assays, 40-fold excess of unlabeled DNA fragments were added to the reaction mixture before adding FAM-labeled probes, and the labeled PARP1-*FoxO1* complexes were almost completely displaced. **c** RD-ES, RD/KO1, and RD/KO1-WT cells were subjected to ChIP analyses using the antibody against PARP1 and an isotype-matched IgG as a negative control. The association of PARP1 with the *FoxO1* gene promoter was quantified by RT-qPCR using primers targeting *FoxO1-L, -M,* and *-R*, respectively. Error bars represent the SD. **d** Identification of PARP1-protected regions on *FoxO1-L* and *FoxO1-R* by DNase I footprinting assays. Electropherograms showed the whole region of the *FoxO1-L/R* after digestion with DNase I following incubation in the presence (blue) or absence (red) of PARP1. The DNA sequences of the PARP1-protected regions were marked with dashed rectangles and denoted as *FoxO1-L-B* (^−813^TCACTGTATTCTT^−826^) and *FoxO1-R-B* (^−1805^TCTTGTGGTCTCTTCACGTTTAC^−1828^).

To demonstrate how PARP1 regulates *FoxO1* transcription, we evaluated whether PARP1 binds to the *FoxO1* promoter. To conduct the EMSA assay, we used three FAM-labeled fragments correspondingly located at −753 to −1032 (*FoxO1-L*), −1289 to −1565 (*FoxO1-M*), and −1678 to −1995 (*FoxO1-R*) upstream of the transcription start site (TSS) in the *FoxO1* promoter region as probes (Fig. 3a; the 2 kb promoter region of the human *FoxO1* gene and the location and sequence of *FoxO1-L*, *FoxO1-M,* and *FoxO1-R* were shown in Supplementary Table S3). The result showed a clear probe band in the control group (Lane 1, no PARP1 for each panel in Fig. 3b). As PARP1 was added by increasing amounts of 2 to 10 μg, the probe band for each group was progressively reduced in size and finally disappeared while more DNA-protein complexes formed (Lanes 2–4; 2, 5, and 10 μg PARP1 for each panel in Fig. 3b). Importantly, adding an excess of 40-fold of unlabeled cold probe DNA partially recovered the probe band and almost completely eliminated FAM-labeled DNA-protein complexes (Lane 5; 10 μg PARP1 for each panel in Fig. 3b). The results indicate that PARP1 specifically binds to DNA sequences that consist of the *FoxO1* promoter region in the in vitro system.

To confirm the in vitro data, we conducted a ChIP assay using RD-ES, RD/KO1 and RD/KO1-WT cells. The results showed that endogenous PARP1 protein bound to the *FoxO1* promoter at the L, M, and R regions in RD-ES and RD/KO1-WT cells, while PARP1 binding in RD/KO1 cells was comparable to that of IgG in the same regions of the *FoxO1* promoter in all tested cells (Fig. 3c). These data further strengthen the conclusion that PARP1 can specifically bind to the *FoxO1* promoter.

To more accurately define the DNA sequences to which PARP1 binds in the *FoxO1* promoter, we performed DNase I footprinting assays with purified PARP1 protein and DNA fragments (L, M, and R) corresponding to the *FoxO1* promoter. The results showed that 30 μg PARP1 produced stronger protection against the DNase I-mediated degradation of L and R than M [Fig. 3d (the indicated sequences) and Supplementary Figure S1]. Two regions (denoted as *FoxO1-L-B* and *FoxO1-R-B*) that seemed to be protected were located at −813 bp to −826 bp and −1805 bp to −1828 bp on the *FoxO1* promoter (Fig. 3d). The results indicate that PARP1 is likely to bind to the *FoxO1* promoter *via* these two regions and regulate *FoxO1* transcription.

### The binding of PARP1 to the *FoxO1* promoter is sequence-specific

**Fig. 4:**
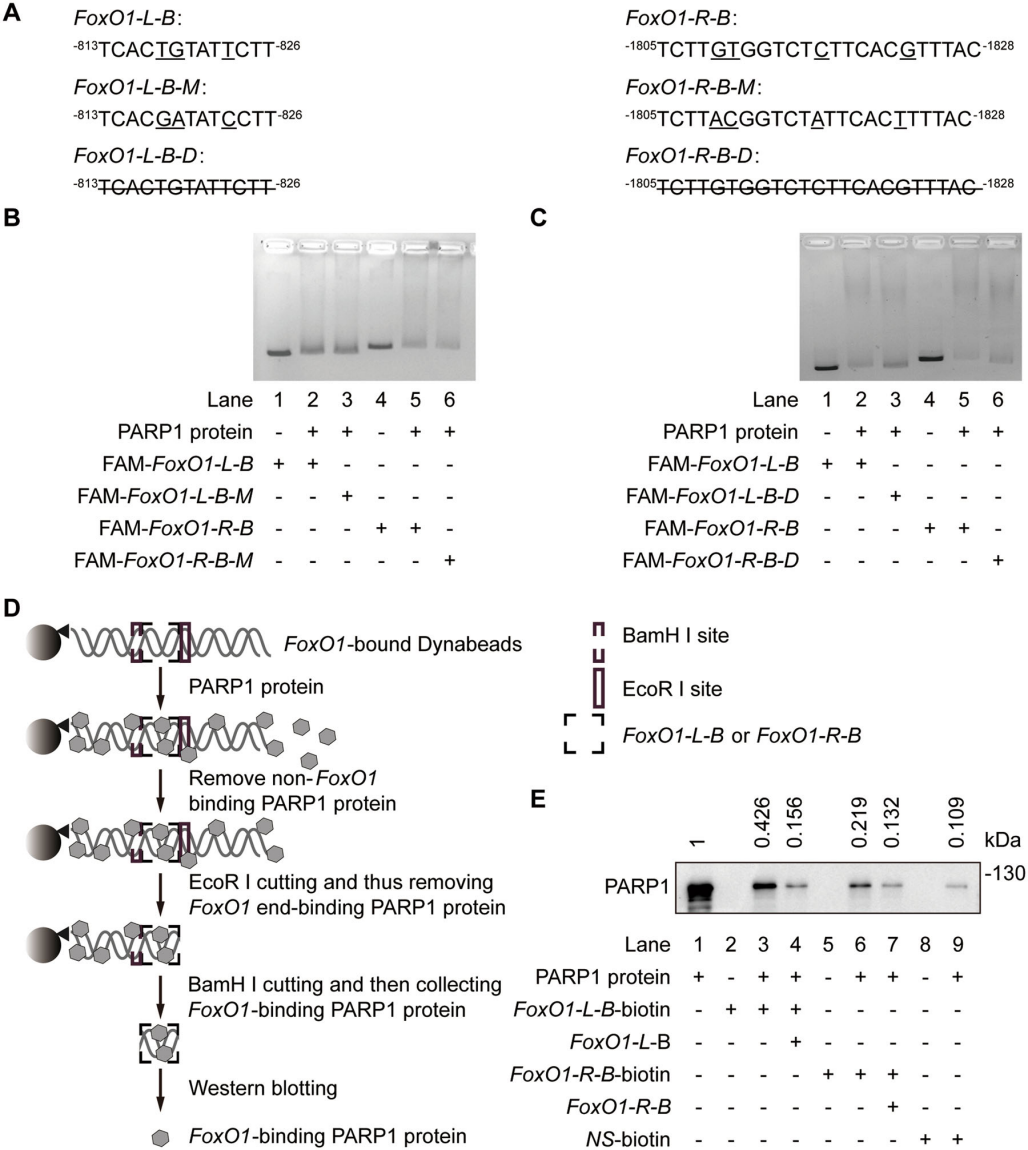
PARP1 binding to the specific DNA sequences on the *FoxO1* promoter. **a** The FAM-labeled probes containing sequences of *FoxO1-L-B* (left) and *FoxO1-R-B* (right) and corresponding mutated probes (underlined; *FoxO1-L-B-M* and *FoxO1-R-B-M*), and the deleted sequences (line-through; *FoxO1-L-B-D* and *FoxO1-R-B-D*). **b** EMSA was carried out using normal or mutated FAM-labeled probes. The amount of DNA-protein complexes detected in FAM-labeled mutant probes was similar to that in FAM-labeled normal probes. **c** EMSA was carried out using FAM-labeled normal and deletion probes. Fewer DNA-protein complexes were detected with FAM-labeled deletion probes than the FAM-labeled normal probes followed by PARP1 incubation. **d** Schematic representation of the flanking restriction enhanced pulldown (FREP). A biotinylated DNA fragment is conjugated to streptavidin-coated magnetic Dynabeads (Invitrogen, Carlsbad, CA). This fragment is engineered to include the *FoxO1-L-B* or *FoxO1-R-B* specific (“bait”) sequence (black dashed box), flanked by restriction enzyme cleavage sites for BamH I proximally (gray dashed box) and EcoR I distally (gray box). DNA-beads are mixed with PARP1 protein. A free non-biotinylated *FoxO1-L-B* or *FoxO1-R-B* DNA fragment can be included in the control reaction at this stage as a specific competitor. Magnetic separation and wash remove non-DNA binding PARP1 protein. EcoR I digestion releases 3′ DNA end-binding PARP1, and BamH I digestion separates the sequence-specific *FoxO1-L-B* or *FoxO1-R-B* binding PARP1 from the 5′ DNA and Dynabeads. Western blotting identifies PARP1 binding to *FoxO1-L-B* or *FoxO1-R-B*. **e** The PARP1-DNA complexes cut with EcoR I and BamH I were detected by western blotting. The relative levels of *FoxO1*-bound PARP1 were presented as the ratio of *FoxO1*-bound PARP1 band intensity/PARP1 input band intensity when the value of PARP1 input band intensity was normalized as l. Lane 1: PARP1 inputs, lane 2: labeled *FoxO1-L-B*-beads, lane 3: PARP1 with labeled *FoxO1-L-B*-beads, lane 4: PARP1 with labeled *FoxO1-L-B*-beads and cold competitor (40-fold excess of free *FoxO1-L-B*-beads), lane 5: labeled *FoxO1-R-B*-beads, lane 6: PARP1 with labeled *FoxO1-R-B*-beads, lane 7: PARP1 with labeled *FoxO1-R-B*-beads and cold competitor (40-fold excess of free *FoxO1-R-B*-beads), lane 8: a labeled non-specific DNA sequence (*NS*-beads) and lane 9: PARP1 with labeled *NS*-beads.

As a DNA repair factor, PARP1 binds to single or double-strand broken DNA without any apparent sequence preference^22,23^. To demonstrate whether PARP1 binding to the *FoxO1* promoter has sequence specificity, we used probes containing *FoxO1-L-B* and *FoxO1-R-B* and their respective mutants containing three or four point mutations (Fig. 4a) as probes in an EMSA assay. Data showed that these point mutations did not cause detectable changes in PARP1 binding to the probe DNA (Lane 3 vs Lane 2 and Lane 6 vs Lane 5 in Fig. 4b). In contrast, complete deletions of *FoxO1-L-B* and *FoxO1-R-B* (Fig. 4a) increased unbound probes (Lane 3 vs Lane 2 and Lane 6 vs Lane 5 in Fig. 4c) probably due to PARP1 binding to the probe DNA. However, these deletions did not fully restore unbound probes to control levels (Lane 3 vs Lane 1 and Lane 6 vs Lane 1 in Fig. 4c), revealing PARP1 non-specific binding, probably because the probes had 2 ends comparable to broken DNA. Nevertheless, the results reveal that PARP1 indeed binds to the *FoxO1* promoter at the −813 to −826 bp (*FoxO1-L-B*) and −1805 to −1828 bp (*FoxO1-R-B*) regions in a sequence-specific manner.

Our conclusion was further supported by data from a novel DNA pulldown assay termed FREP that was done to minimize detectable non-specific PARP1 binding through restriction enzyme digestion^24^. To do this assay, the 3′ free end of the DNA was cleaved off by EcoR I and the single-stranded DNA that was not cleaved off with BamH I was therefore discarded along with the bead (Fig. 4d). *FoxO1-L-B* and *FoxO1-R-B* were used as corresponding competitors of the biotin-labeled *FoxO1-L-B* and *FoxO1-R-B*, while a non-specific 31 bp DNA sequence (*NS*) labeled with biotin was used as the control for non-specific PARP1 binding. The PARP1-DNA complexes digested with EcoR I and BamH I were detected by western blotting with an anti-PARP1 antibody. Results (Fig. 4e) revealed that PARP1 bound to the biotin-labeled *FoxO1-L-B* (Lane 3) and *FoxO1-R-B* (Lane 6) much more than the control (Lane 9). Importantly, PARP1 bound to biotin-labeled *FoxO1-L-B* and *FoxO1-R-B* was largely competed away by free *FoxO1-L-B* and *FoxO1-R-B*, respectively (Lane 4 and 7, Fig. 4e). Therefore, these data further indicate that PARP1 sequence-specific binding to the *FoxO1* promoter inhibits *FoxO1* transcription.

### PARP1 transcriptional regulation of *FoxO1* is independent of its catalytic activity

**Fig. 5:**
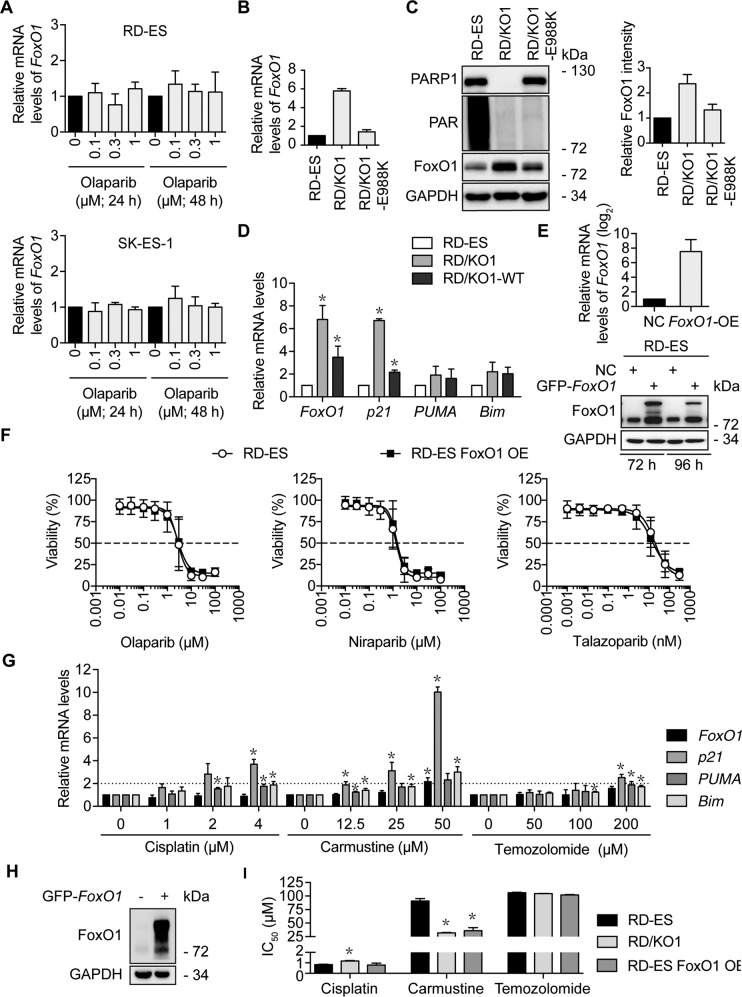
The expression of *FoxO1* regulated by PARP1 is independent of its catalytic activity and FoxO1 does not affect the sensitivity of RD-ES cells to PARP inhibitors. **a** RD-ES (upper) and SK-ES-1 (lower) cells were incubated in the indicated concentrations of olaparib for 24 h or 48 h. Then, mRNA levels of *FoxO1* were detected by RT-qPCR. **b** mRNA levels of *FoxO1* in RD/KO1 cells and their stably-transfected with mutated-*PARP1* cDNA (E988K) variants were detected by RT-qPCR. **c** Protein levels of FoxO1 in RD/KO1 cells and their stably-transfected with mutated-*PARP1* cDNA (E988K) variants were detected by western blotting. The relative FoxO1 levels were presented as the ratio of (FoxO1/GAPDH)_KO1 or E988K_/(FoxO1/GAPDH)_RD-ES_ when the value of (FoxO1/GAPDH)_RD-ES_ was normalized as l. Data were expressed as mean ± SD from three independent experiments. **d** Effects of *PARP1* loss and *PARP1* reconstitution on the expression of FoxO1 target genes. mRNA levels were detected by RT-qPCR. *, *p* < 0.05. **e** RD-ES cells were transfected with GFP-*FoxO1* cDNA for 72 h and 96 h, and the mRNA (left) and protein (right) levels of FoxO1 were detected by RT-qPCR and western blotting, respectively. **f** Survival curves of olaparib, niraparib and talazoparib-treated RD-ES and *FoxO1*-overexpressed RD-ES (RD-ES FoxO1 OE) cells assessed by CCK-8 assays. Error bars represent the SD. **g** Changes of the mRNA levels of *FoxO1* and its target genes. RD-ES cells were treated with cisplatin, carmustine and temozolomide for 12 h. Then, mRNA levels of *FoxO1*, *p21*, *PUMA* and *Bim* were detected by RT-qPCR and normalized to those in RD-ES cells without any treatments. *, *p* < 0.05. **h** FoxO1 overexpression by transfecting GFP-*FoxO1* into RD-ES cells was determined by western blotting. **i** Sensitivity of RD-ES, RD/KO1 and RD-ES FoxO1 OE cells to cisplatin, carmustine and temozolomide. Cells were exposed to gradient concentrations of the tested agents for 72 h. IC_50_ values from three independent experiments were expressed as mean ± SD. Error bars represent the SD. *, *p* < 0.05.

PARP1 has been shown to regulate gene transcription in two modes independent of or dependent on its catalytic activity^15^. To test whether its poly(ADP-ribose) polymerase activity is required for its transcriptional regulation on *FoxO1*, we treated RD-ES and SK-ES-1 cells with the PARPi olaparib. Olaparib inhibits the PARP1 enzymatic activity but does not affect PARP1 expression^3,25^. The treatments with olaparib did not cause obvious changes in mRNA or protein levels of *FoxO1* (Fig. 5a and Supplementary Fig. S2). To verify this result, we used a catalytic mutant of PARP1 by replacing the glutamic acid residue at 988 with a lysine residue (E988K) to complement RD/KO1 (resulting cells, RD/KO1-E988K)^4^. E988K has no poly(ADP-ribose) polymerase activity but keeps the mono-ADP-ribosyl-transferase activity^4^. The expression of E988K in RD-ES/KO1 cells led to apparent decreases in *FoxO1* mRNA and protein levels (Fig. 5b, c). These results further indicate that transcriptional regulation of *FoxO1* by PARP1 is independent of its catalytic activity.

### FoxO1 does not contribute to the sensitivity of RD-ES cells to PARP inhibitors

We further evaluated the effects of PARP1 loss and reconstitution on the target genes of the transcription factor FoxO1. The data revealed that loss and exogenous re-expression of *PARP1* in RD-ES cells caused similar significant changes in mRNA levels of *FoxO1* and *p21*, one of the target genes of FoxO1^26^. However, only marginal changes took place in mRNA levels of *PUMA* and *Bim*, the other two target genes of FoxO1^27^ (Fig. 5d).

We demonstrate that *PARP1* loss leads to FoxO1 overexpression and cellular resistance to PARP inhibitors. Therefore, we further investigated whether FoxO1 overexpression contributed to PARPi resistance. We established a FoxO1-overexpressed model by transfecting GFP-*FoxO1* into RD-ES cells that normally express PARP1 protein. FoxO1 overexpression was verified in Fig. 5e. However, FoxO1 overexpression did not change the cellular sensitivity of PARPis olaparib, niraparib, or talazoparib (Fig. 5f), indicating that there is no correlation between FoxO1 expression and the sensitivity of RD-ES cells to PARPis.

FoxO1 has been shown to regulate cellular sensitivity to several DNA damaging agents^28^. We thus evaluated the changes in the mRNA levels of *FoxO1* and its target genes in RD-ES cells exposed to cisplatin, carmustine, and temozolomide. As shown in Fig. 5g, only carmustine increased the mRNA levels of *FoxO1* at its high concentration. Notably, changes in the mRNA levels of *p21*, *PUMA*, and *Bim* were different in response to different treatments.

Then we tested the sensitivity of RD-ES, RD/KO1, and *FoxO1*-overexpressed RD-ES (RD-ES FoxO1 OE) (Fig. 5h) cells to cisplatin, carmustine, and temozolomide. The results suggested that the decreased sensitivity of RD/KO1 cells to cisplatin was probably not caused by increased FoxO1 expression. In contrast, the sensitivity of both RD/KO1 and RD-ES FoxO1 OE cells to carmustine significantly increased while the sensitivity of these cells to temozolomide basically kept unchanged relative to that of RD-ES cells (Fig. 5i). Carmustine but not the other two alkylating agents showed some dependency on FoxO1 in its killing RD-ES, which was consistent with the fact that cisplatin and temozolomide did not cause significant changes in the mRNA levels of *FoxO1* while carmustine could significantly increase the mRNA levels of *FoxO1* at 50 μM (Fig. 5g).

## Discussion

Recent studies on PARP1 have been largely focused on its functions in DNA repair, primarily due to the successful clinical uses of PARPis. This may be a reason why our understanding on PARP1 transcriptional regulation has been less established. In this study, we investigated PARP1-mediated gene transcription by using *PARP1*-KO cells which did not respond to PARPi treatments. *FoxO1*, encoding a transcription factor, was up-regulated in its mRNA and protein levels in the *PARP1*-KO Ewing sarcoma RD/KO1, Cri/KO, and SK/KO1 cells and pancreatic cancer CAPAN1/KO cells. Importantly, *PARP1* complementation prevented *PARP1* loss-mediated increase in its mRNA or protein levels, though partly in some cells. The results indicate that PARP1 represses *FoxO1* gene transcription. We subsequently demonstrated that PARP1 specifically bound to the *FoxO1* promoter. It was also revealed that regions of −813 to −826 bp (i.e., 5′-TCACTGTATTCTT-3′) and −1805 to −1828 bp (i.e., 5′-TCTTGTGGTCTCTTCACGTTTAC-3′) upstream of the TSS on the *FoxO1* promoter were required for this binding, indicating its DNA sequence dependency or specificity. Moreover, negative transcriptional regulation of *FoxO1* by PARP1 was independent of its enzymatic activity. This may be why *FoxO1* expression is not correlated with cellular PARPi sensitivity because almost all the present PARPis are enzymatic inhibitors. Nevertheless, our data suggest a possible correlation of the transcriptional regulation of *FoxO1* by PARP1 with the carmustine sensitivity in RD-ES cells. This provides a direction for our future exploration.

As a DNA repair factor, PARP1 can bind to DNA in a DNA sequence-independent manner, and inhibition of its enzymatic activity increases this binding^4,23,29^. In striking contrast, when functioning as a transcriptional regulation factor, the binding of PARP1 to gene promoter regions requires particular DNA sequences. For example, PARP1 was shown to bind to the 5′-GTTTCACAAT-3′ sequence in the *BRCA2* promoter^14^, to the 5′-GCTGTGGGAA-3′ sequence in the *Tcirg1* promoter^29^, to the 5′-ATGGTcttACCTA-3′ sequence in the *HFE* promoter^30^, to the 5′-GTTG-3′ sequence in the *CXCL1* promoter^31^, and to the 5′-TGTTG-3′ sequence in the *cTnT* promoter^32^. The PARP1 binding results in negative transcriptional regulation of the former three genes but positive regulation of the latter two. Different from only a single-nucleotide sequence motif that is required for the PARP1 binding in these studies^14,29–32^, our data reveal that two specific motifs (i.e., 5′-TCACTGTATTCTT-3′ and 5′-TCTTGTGGTCTCTTCACGTTTAC-3′) are needed for *FoxO1* transcriptional inhibition. The two motifs are separately located at the regions at a distance of 979 bp on the *FoxO1* promoter. This is a new PARP1-gene promoter binding mode. Notably, the 5′-TG-3′ nucleotide sequence appears in the above mentioned PARP1 binding motifs (except *BRCA2*). However, this sequence might not be a critical consensus nucleotide sequence for the PARP1-gene promoter binding because our result revealed that the mutation of 5′-TG-3′ did not change the PARP1 binding. Therefore, previous studies and our own indicate that transcriptional regulation by PARP1, at least by way of nucleotide-sequence-dependent binding to the gene promoter, is gene specific in the aspects of PARP1-bound DNA sequence(s), transcriptional inhibition or stimulation and PARP1-polymerase dependency. At present, the issues on this specificity remain to be explored, particularly including what the determining factors are, whether any other cofactors are involved, and which domain(s) of PARP1 contribute to specific binding.

The transcription factor FoxO1 is a member of the FoxO family and regulates diverse gene expression in controlling various biological processes such as tumorigenesis and aging^33^. FoxO1 can function as a tumor suppressor. On the one hand, FoxO1 inhibits cancer cell proliferation by activating the transcription of *p21*, encoding a cell cycle inhibitor^26^; on the other, FoxO1 induces apoptosis by upregulating expression of several pro-apoptotic factors including *PUMA* and *Bim*^26,34^. Moreover, FoxO1 also affects cellular sensitivity or resistance to anticancer drugs^26,28,34–36^. Though its post-transcriptional modifications and its transcriptional control of target genes have been extensively investigated^37^, relatively little is known about transcriptional regulation of the *FoxO1* gene itself. Our current study reveals that PARP1 binds to the *FoxO1* promoter and represses *FoxO1* expression, which is independent of PARP1 enzymatic activity. Knockout and complementation of *PARP1* separately caused a consistent increase and reduction in both mRNA and protein levels of FoxO1. Notably, cells that normally express PARP1 and that are complemented with *PARP1* (WT or mutated) have low levels of *FoxO1* expression. Therefore, PARP1 binding appears not to affect the basal expression of the *FoxO1* gene. This conclusion might be supported also by the finding that in HEK293T cells transfected with both FLAG-*PARP1* and HA-*FoxO1*, both proteins can be detected at the same time^38^. Therefore, in addition to transcription factors E2F-1^39^, FoxC1^40^, FoxO3^41^, and EWS-FLI1^21^, PARP1 is another new transcriptional regulator of the *FoxO1* gene by direct binding to its promoter.

Collectively, this study demonstrates a new PARP1-gene promoter binding mode evidenced by direct PARP1 binding to two separate motifs on the *FoxO1* promoter. PARP1 is a new transcriptional repressor of *FoxO1*, encoding an important transcription factor with extensive biological functions. The regulation of *FoxO1* expression by PARP1 is independent of its polymerase activity and cellular PARPi sensitivity. These findings provide new insights into both PARP1 functions and *FoxO1* transcriptional regulation, helping to further understand the roles of PARP1 and FoxO1 in tumorigenesis and cancer therapy.

## Materials and methods

Details about drugs and antibodies, cell culture, stable KO of PARP1 with the CRISPR/Cas9 technique, RNA sequencing (RNA-seq), quantitative real-time polymerase chain reaction (RT-qPCR), EMSA and competitive binding assays, ChIP, DNase I footprinting assays, and FREP are provided in Supplementary Materials and Methods.

### Western blotting

Standard western blotting^42^ was used to detect the changes in protein levels caused by the indicated treatments.

### Cytotoxicity assays

Cell Counting Kit 8 (CCK-8, Dojindo Laboratories, Kumamoto, Japan) assays were used to detect cytotoxicity as previously described^43^.

### Plasmid construction, PARP1 protein purification, generation of cells expressing PARP1 or its mutants

Plasmid construction, PARP1 protein purification, generation of cells expressing PARP1, or its mutants were conducted as previously reported^4^.

### Statistical analysis

All data were representative of three independent experiments. Data were presented as mean ± SD (standard deviation), and no data was excluded from analysis. A Student’s *t* test (two-tailed) was used to compare two groups. Only *p* < 0.05 was considered statistically significant.

## Supplementary information

Supplementary Materials and Methods

Supplementary Figure Legends

Supplementary Fig. S1

Supplementary Fig. S2

Supplementary Table Legends

Supplementary Table S1

Supplementary Table S2

Supplementary Table S3

Supplementary Table S4

Declaration of contribution to article

Reporting Checklist
