# Supplementary material for: Polymerase independent repression of FoxO1 transcription by sequence-specific PARP1 binding to FoxO1 promoter
Source: Cell Death Dis. 2020 Jan 28;11(1):71. doi: 10.1038/s41419-020-2265-y (PMC6987093; doi:10.1038/s41419-020-2265-y)
Supplement: Supplementary file 1 — Supplementary Materials and Methods [file 41419_2020_2265_MOESM1_ESM.doc]

**Polymerase independent repression of *FoxO1* transcription by sequence-specific PARP1 binding to *FoxO1* promoter**

Yu-Nan Tian^1,2^, Hua-Dong Chen^1,2^, Chang-Qing Tian^1,2^, Ying-Qing Wang^1,2^ and Ze-Hong Miao^1,2,3^

# Supplementary Materials and Methods

**Drugs and antibodies**

Olaparib was purchased from LC Laboratories (Woburn, MA, USA). Niraparib and talazoparib were purchased from Selleck Chemicals (Shanghai, China). Cisplatin, carmustine and temozolomide were purchased from MedChemExpress (Shanghai, China). All drugs [except cisplatin was dissolved in dimethylformamide (DMF)] were dissolved in 100% dimethyl sulfoxide (DMSO), aliquoted, and stored at -20° C. Upon use, the drugs were diluted to the desired concentrations in normal saline with a final DMSO concentration not exceeding 0.1%.

Antibodies against PARP1 (sc-7150), γH2AX (sc-101696), CHK1 (sc-8408) and RAD51 (sc-8349) were from Santa Cruz Biotechnology (Santa Cruz, CA, USA). Antibodies against RPA32 (#2208), FoxO1 (#2880), A20/TNFAIP3 (#5630S), IκBα (#9242S), NF-κB1 p105/p50 (#13586S) were from Cell Signaling Technology (Danvers, MA, USA). The antibody against CHK2 (ab109413) was from Abcam (Cambridge, UK). The anti-PAR polyclonal antibody (4336-BPC-100) was from Trevigen (Gaithersburg, MD, USA). The anti-GAPDH mouse monoclonal antibody (AG019) was purchased from Beyotime (Shanghai, China). The secondary antibodies used for western blotting were HRP-conjugated goat anti-rabbit and goat anti-mouse antibodies (Jackson Immuno Research Laboratories Inc., West Grove, PA, USA).

# Cell culture

Human Ewing sarcoma RD-ES and SK-ES-1 cells and pancreatic cancer CAPAN1 cells were purchased from American Type Culture Collection (ATCC, Manassas, VA, USA). RD-ES cells were cultured in RPMI 1640 (Invitrogen, Carlsbad, CA, USA) supplemented with 15% fetal bovine serum (FBS). SK-ES-1 cells were grown in McCoy’s 5A media supplemented with 15% FBS, and CAPAN1 cells were cultured in IMDM containing 10% FBS. Short tandem repeat (STR) profiles were determined for each cell line using the Promega PowerPlex 16 System by Shanghai Genesky Bio-Tech CO., LTD. Cells were also periodically authenticated with morphologic inspection and tested for mycoplasma contamination. All cell lines were maintained at 37° C in 5% CO_2_. *PARP1*-KO cells were generated by TALEN-mediated gene targeting as previously reported^1^.

# Stable knockout of *PARP1* with the CRISPR/Cas9 technique

# Lentiviral transfection of cultured cells with pLentiCRISPRv2 vectors encoding *PARP1*-specific CRISPR or control vectors (Obio Technology, Shanghai, China) were performed according to the supplier’s instructions. The oligonucleotide single guide RNA sequence for *PARP1* was 5' -CACCGCTTGGGACCGGGATTTCATC-3'. Transduced CAPAN1 and RD-ES cells were selected with 1 μg/mL puromycin for 2 weeks, and downregulated PARP1 expression was identified by western blotting analysis. The resulting cells were denoted as CAPAN1/KO and Cri/KO cells, respectively derived from CAPAN1 and RD-ES cells.

# Cytotoxicity assays

RD-ES and SK-ES-1 cells were exposed to gradient concentrations of drugs for 72 h. Cell viability was assessed using a Cell Counting Kit 8 assay (CCK-8, Dojindo Laboratories, Kumamoto, Japan) and recorded with a spectra-MAX190 (Molecular Devices, San Jose, CA, USA) as previously described^2^. The proliferative inhibition rate (%) was calculated as [1-(A450_treated_/A450_control_)] × 100%. The averaged IC_50_ values were determined with the Logit method from three independent experiments.

# RNA sequencing (RNA-seq)

Cells were harvested in TRIzol (Life Technologies; CA, USA) and extracted for RNA. The mRNA was enriched with oligo (dT) and fragmented into small fragments. These fragments underwent reverse transcription, 3' and 5' adaptors ligation, PCR amplification, library construction, RNA sequencing using HiSeq 2500 (Illumina Inc., San Diego, CA, USA) and data analysis at Genergy Biotechnology Co., Ltd. (Shanghai, China).

# Quantitative real-time polymerase chain reaction (RT-qPCR)

Total RNA was extracted using TRIzol (Invitrogen, Carlsbad, CA, USA). The cDNA was generated using a reverse transcription reagent kit (TaKaRa, Tokyo, Japan, USA). RT-qPCR reactions were performed using a 7500 Fast Real-time PCR System (Applied Biosystem, Grand Island, NY, USA). All experiments were performed in triplicate and were normalized to *β-Actin* transcript levels with PCR system software. The primers used are listed in supplementary Table S4.

# Electrophoretic mobility shift (EMSA) and competitive binding assays

The DNA-protein interaction was detected using Light-Shift^TM^ Chemiluminescent EMSA kit (Thermo Fisher Scientific, Waltham, MA, USA) according to the manufacturer’s protocol. The sequence of *FoxO1* oligonucleotides was: For *FoxO1-L*, CTAGCGTTTAAACTTAAGCTTAGCCTGTGCCATTCGGTCTA and AACGGGCCCTCTAGACTCGAGTGGTTCTCTCTAACTGCGCTC; for *FoxO1-M*, CTAGCGTTTAAACTTAAGCTTAGGTGGGAAGATAATGGCCC and AACGGGCCCTCTAGACTCGAGACAACCTCTTGTCCAACTGACT; and for *FoxO1-R*, CTAGCGTTTAAACTTAAGCTTTAGTCCGGGCTCCTGTTTCT and AACGGGCCCTCTAGACTCGAGTTTTAGGGTGCGGCTGTCT.

The *FoxO1* promoter regions were amplified from RD-ES genomic DNA and cloned into a pcDNA3.1(+) vector. Mutation mutagenesis of *FoxO1-L-B* and *FoxO1-R-B* (labeled as *FoxO1-L-B-M* and *FoxO1-R-B-M*) and deletion mutagenesis of *FoxO1-L-B* and *FoxO1-R-B* (labeled as *FoxO1-L-B-D* and *FoxO1-R-B-D*) were generated by using a muta-direct site-directed mutagenesis kit (Beijing SBS Genetech Co, Ltd, Beijing, China) according to the manufacturer’s instructions and confirmed by DNA sequencing. To prepare fluorescent 6-carboxy-fluorescein (FAM) labeled probes, the promoter regions were amplified by PCR from the carrier pcDNA3.1 (+) using primers of T7 (FAM) and pcDNA3.1R. The FAM-labeled probes were purified with the Wizard® SV Gel and PCR Clean-Up System (Promega, Fitchburg, WI, USA) and quantified with NanoDrop 2000C (Thermo Fisher Scientific, Waltham, MA, USA). For EMSA reactions, different amounts of PARP1 protein (0, 2, 5 or 10 µg) were pre-incubated with 50 ng DNA probe for 30 min. After incubation, DNA-protein complexes and free DNA were separated by electrophoresis in nondenaturing polyacrylamide gels. The competitive EMSA was performed similarly, except that 2 µg of unlabeled *FoxO1-L-B* or *FoxO1-R-B* were incubated simultaneously with 50 ng of FAM-*FoxO1-L-B* or FAM-*FoxO1-R-B*.

# Chromatin immunoprecipitation (ChIP)

# The ChIP assay was performed using a simpleChIP plus enzymatic chromatin IP kit (#9003, Cell Signaling Technology, Danvers, MA, USA) according to the procedure provided by the manufacturer. Cells were cross-linked using 1% formaldehyde and fragmented with sonication, followed by immunoprecipitation. The cross-linked DNA was reversed and purified, and the endogenous association of PARP1 with *FoxO1* was detected by RT-qPCR using primers located in L, M and R regions. Antibodies used for ChIP were anti-PARP1 (ab227244, Abcam, Cambridge, UK) and anti-histone H3 (#4620, Cell Signaling Technology, Danvers, MA, USA). The final ChIP DNA was then used as templates in RT-qPCR reactions, using primers targeting the *FoxO1* promoter, and primer sequences are attached in the Supplementary Table S4.

# DNase I footprinting assays

DNase I footprinting assays were performed as described previously^3^. For each assay, 400 ng probes were incubated with different amounts of PARP1 in a total volume of 40 μl. After incubating 30 min at 25° C, 10 μl of solution containing 0.015 units of DNase I (Promega, Fitchburg, WI, USA) and 100 nmol freshly prepared CaCl_2_ was added and further incubated for 1 min at 25° C. The reaction was stopped by adding 140 μl of DNase I stop solution (200 mM unbuffered sodium acetate, 30 mM EDTA and 0.15% SDS). Samples were first extracted with phenol/chloroform and then precipitated with ethanol. The pellets were dissolved in 30 μl of MiniQ water. The preparation of the DNA ladder, electrophoresis and data analysis were the same as described previously^4^, except that the GeneScan-LIZ500 size standard (Applied Biosystem, Grand Island, NY, USA) was used.

# Flanking restriction enhanced pulldown (FREP)

FREP is a novel DNA pulldown method that reduces non-specific binding to the DNA probe through sequential enzymatic restriction. FREP assays were performed as previously described^5,6^ with small modifications. Bead-linked DNA was mixed with 1 μg of PARP1 protein in 1x binding buffer (10 mM Tris pH 7.5, 50 mM KCl, 5mM MgCl_2_, 2.5% glycerol, 0.5% NP-40, and 1 μg of polydI-dC) at room temperature for 2 h. After magnetic selection and washing with PBS + 0.05% Tween 20, DNA beads with bound proteins were digested with EcoR I at 37° C for 3 h. After another magnetic selection and washing, the DNA beads were digested with BamH I at 37°C for 1 h, and the supernatant was collected for western blotting analyses. The “bait” DNA fragments used for FREP are attached in the Supplementary Table S4.

**References**

1. Chen, H. D. et al. Increased PARP1-DNA binding due to autoPARylation inhibition of PARP1 on DNA rather than PARP1-DNA trapping is correlated with PARP1 inhibitor's cytotoxicity. *Int. J. Cancer* **145**, 714-727 (2019).

2. Wang, W. et al. MCL-1 degradation mediated by JNK activation via MEKK1/TAK1-MKK4 contributes to anticancer activity of new tubulin inhibitor MT189. *Mol. Cancer Ther.* **13**, 1480-1491 (2014).

3. Wang, Y., Cen, X. F., Zhao, G. P. & Wang, J. Characterization of a new GlnR binding box in the promoter of amtB in Streptomyces coelicolor inferred a PhoP/GlnR competitive binding mechanism for transcriptional regulation of amtB. *J. Bacteriol.* **194**, 5237-5244 (2012).

4. Wu, K. F. et al. CpsR, a GntR family regulator, transcriptionally regulates capsular polysaccharide biosynthesis and governs bacterial virulence in Streptococcus pneumoniae. *Sci. Rep.* **6** (2016).

5. Li, G. et al. The rheumatoid arthritis risk variant CCR6DNP regulates CCR6 via PARP-1. *PLoS Genet.* **12** (2016).

6. Li, G. et al. High-throughput identification of noncoding functional SNPs via type IIS enzyme restriction. *Nat. Genet.* **50**, 1180-1188 (2018).
