# Supplementary material for: Polymerase independent repression of FoxO1 transcription by sequence-specific PARP1 binding to FoxO1 promoter
Source: Cell Death Dis. 2020 Jan 28;11(1):71. doi: 10.1038/s41419-020-2265-y (PMC6987093; doi:10.1038/s41419-020-2265-y)
Supplement: Supplementary file 2 — Supplementary Figure Legends [file 41419_2020_2265_MOESM2_ESM.doc]

**Polymerase independent repression of *FoxO1* transcription by sequence-specific PARP1 binding to *FoxO1* promoter**

Yu-Nan Tian^1,2^, Hua-Dong Chen^1,2^, Chang-Qing Tian^1,2^, Ying-Qing Wang^1,2^ and Ze-Hong Miao^1,2,3^

**Supplementary Figure Legends**

# Supplementary Figure S1. Identification of PARP1-protected regions on *FoxO1-M* by DNase I footprinting assays. Electropherograms showed the whole region of the *FoxO1-M* after digestion with DNase I following incubation in the presence (blue) or absence (red) of PARP1.

# Supplementary Figure S2. No change in FoxO1 protein levels from exposure to PARPi olaparib. **RD-ES and SK-ES-1 cells were incubated in the indicated concentrations of olaparib for 24 h or 48 h. Then, the protein level of FoxO1 was detected by western blotting.**
