# Supplementary figures and images for: Polymerase independent repression of FoxO1 transcription by sequence-specific PARP1 binding to FoxO1 promoter
Source: Cell Death Dis. 2020 Jan 28;11(1):71. doi: 10.1038/s41419-020-2265-y (PMC6987093; doi:10.1038/s41419-020-2265-y)

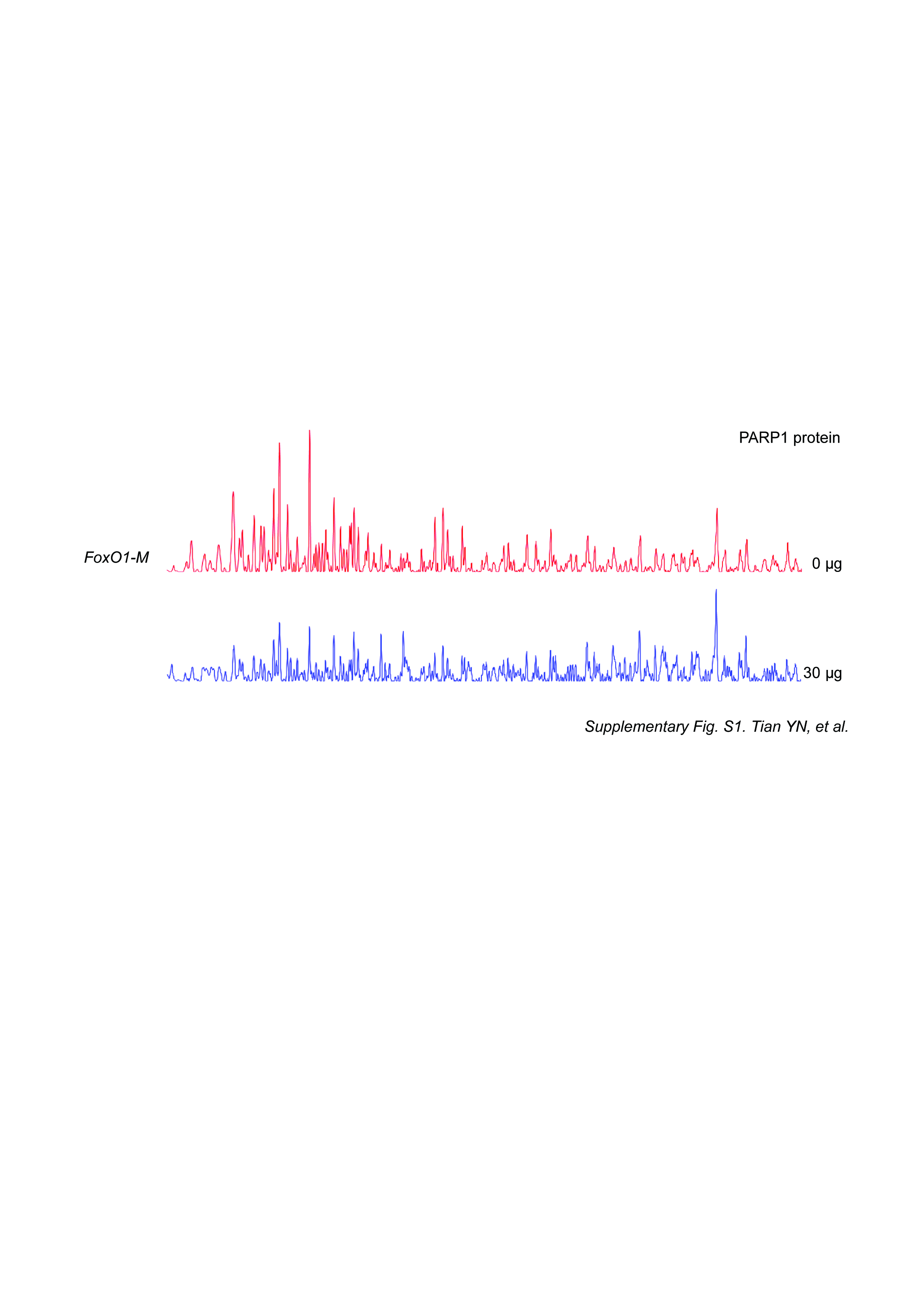

Supplement: Supplementary file 3 — Supplementary Fig. S1 [file 41419_2020_2265_MOESM3_ESM.tif]

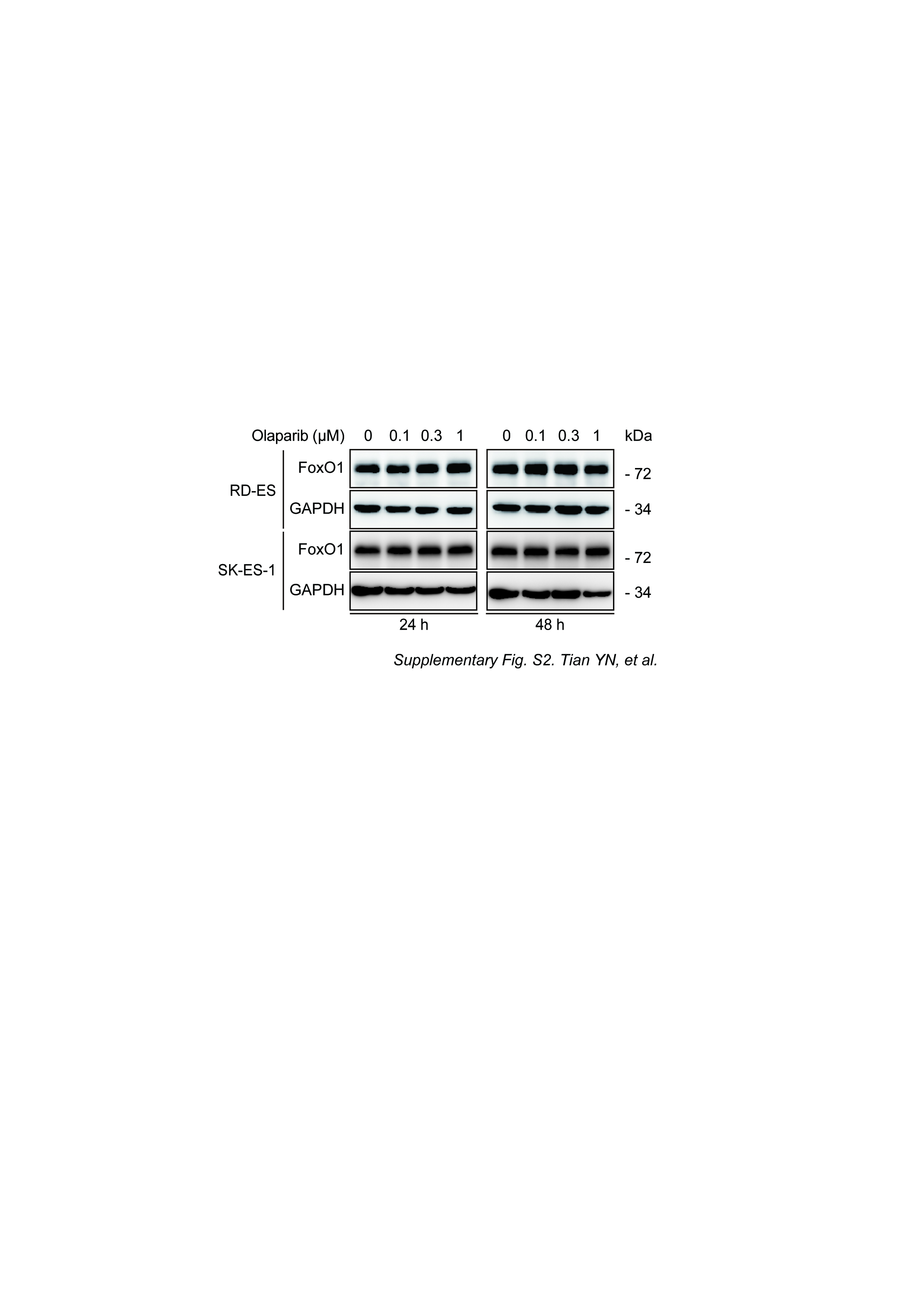

Supplement: Supplementary file 4 — Supplementary Fig. S2 [file 41419_2020_2265_MOESM4_ESM.tif]
