# Supplementary material for: Polymerase independent repression of FoxO1 transcription by sequence-specific PARP1 binding to FoxO1 promoter
Source: Cell Death Dis. 2020 Jan 28;11(1):71. doi: 10.1038/s41419-020-2265-y (PMC6987093; doi:10.1038/s41419-020-2265-y)
Supplement: Supplementary file 5 — Supplementary Table Legends [file 41419_2020_2265_MOESM5_ESM.doc]

**Polymerase independent repression of *FoxO1* transcription by sequence-specific PARP1 binding to *FoxO1* promoter**

Yu-Nan Tian^1,2^, Hua-Dong Chen^1,2^, Chang-Qing Tian^1,2^, Ying-Qing Wang^1,2^ and Ze-Hong Miao^1,2,3^

**Supplementary Table Legends**

**Supplementary Table S1. The list of 492 differentially expressed genes revealed by RNA-Seq assays in RD/KO1 cells.**

Notes:

- Inf : FPKM_RD-ES=0
- #NAME? : FPKM_RD/KO1=0
- Log_2_ Fold Change= Log_2_ (FPKM_RD/KO1) / (FPKM_RD-ES)

The FPKM (Fragments per kilo bases per million reads) value of different genes under different experimental conditions was taken as the expression level. The FPKM was presented as the ratio of (total exon fragments / mapped reads [millions]) / (exon length^1^). The ratio of (total exon fragments / mapped reads [millions]) is the read count mapped to the gene normalized to total read counts. The value is then normalized to gene length (exon length^1^) so that the expression of genes with different sequencing depths and length are comparable.

**Supplementary Table S2. The list of 10 differentially expressed genes involved in both “pathways in cancer” and “regulation of sequence-specific DNA binding transcription factor activity” in RD/KO1 cells.**

**Supplementary Table S3. The nucleotide sequence within 2000 bp from the transcriptional start site on the *FoxO1* promoter and the precise locations and sequences of *FoxO1-L* (yellow), *FoxO1-M* (pink) and *FoxO1-R* (green).** *FoxO1-L-B* contains 13 bp located between -813 and -826 (yellow and bold/underlined), while *FoxO1-R-B* contains 23 bp located between -1805 and -1828 (green and bold/underlined).

**Supplementary Table S4. Primer sequences used in the study.**

**References**

1. Akbari, O. S., Papathanos, P. A., Sandler, J. E., Kennedy, K. & Hay, B. A. Identification of germline transcriptional regulatory elements in Aedes aegypti*. Sci R*e**p** 4, 3954 (2014).
