# Supplementary material for: Polymerase independent repression of FoxO1 transcription by sequence-specific PARP1 binding to FoxO1 promoter
Source: Cell Death Dis. 2020 Jan 28;11(1):71. doi: 10.1038/s41419-020-2265-y (PMC6987093; doi:10.1038/s41419-020-2265-y)
Supplement: Supplementary file 6 — Supplementary Table S1 [file 41419_2020_2265_MOESM6_ESM.doc]

**Supplementary Table S1.**

| **No.** | **Genes** | **Log_2_ Fold Change** | ***p*-value** |
| --- | --- | --- | --- |
| **Up-regulated** |  |  |  |
| 1 | GATA4 | 4.42609 | 0.0113 |
| 2 | IL7R | 4.29414 | 0.00125 |
| 3 | NFKB2 | 3.70595 | 0.01115 |
| 4 | BIRC3 | 4.30811 | 0.0009 |
| 5 | PTN | 3.97956 | 0.0041 |
| 6 | TNFAIP6 | 4.94917 | 0.0273 |
| 7 | PDPN | 3.1843 | 0.00025 |
| 8 | VCAM1 | 5.30955 | 0.02365 |
| 9 | NFKB1 | 3.19954 | 0.00275 |
| 10 | IER3 | 2.7689 | 0.0002 |
| 11 | BNC1 | 3.04941 | 0.02925 |
| 12 | RELB | 3.53061 | 0.0016 |
| 13 | ARHGEF16 | 3.27508 | 0.0434 |
| 14 | HCLS1 | 12.2769 | 0.04965 |
| 15 | NFKBIA | 2.72184 | 0.00005 |
| 16 | ZC3H12A | 3.19401 | 0.001 |
| 17 | EFNA1 | 3.16012 | 0.0296 |
| 18 | LINC01033 | inf | 0.00095 |
| 19 | CLIC2 | 4.10656 | 0.00355 |
| 20 | PERP | 3.4663 | 0.00895 |
| 21 | RAI14 | 3.93783 | 0.04055 |
| 22 | TAPBP | 2.33609 | 0.00005 |
| 23 | CXCL1 | 3.82775 | 0.03925 |
| 24 | ZSCAN9 | 15.6369 | 0.04115 |
| 25 | COL22A1 | 3.51152 | 0.00175 |
| 26 | SCG2 | 3.14079 | 0.00335 |
| 27 | CA8 | inf | 0.00005 |
| 28 | TNFRSF9 | inf | 0.00005 |
| 29 | FAM65C | 3.98049 | 0.00415 |
| 30 | COL7A1 | 3.88425 | 0.00005 |
| 31 | KM-PA-2 | 3.86818 | 0.005 |
| 32 | NFKB2 | 2.68852 | 0.0007 |
| 33 | AC073135.3 | 2.80818 | 0.0239 |
| 34 | HLA-B | 2.5046 | 0.00005 |
| 35 | HDAC9 | 2.85237 | 0.0009 |
| 36 | KIF1A | 4.18513 | 0.00005 |
| 37 | CXCL5 | inf | 0.00005 |
| 38 | CCL2 | 4.98258 | 0.02545 |
| 39 | TNFRSF4 | inf | 0.00005 |
| 40 | PRRX1 | 5.06348 | 0.00505 |
| 41 | CXCL6 | inf | 0.00005 |
| 42 | DRAM1 | 3.79909 | 0.00005 |
| 43 | SELM | 4.6688 | 0.00125 |
| 44 | TNFAIP3 | 5.80766 | 0.00055 |
| 45 | LCP1 | 4.85617 | 0.00005 |
| 46 | LGALS1 | 4.81584 | 0.0004 |
| 47 | LRRC4C | 2.57605 | 0.0232 |
| 48 | DDX58 | 2.58398 | 0.0041 |
| 49 | WNT10A | 4.59847 | 0.0386 |
| 50 | SLC16A9 | 2.80009 | 0.01565 |
| 51 | YPEL5 | 2.07857 | 0.0214 |
| 52 | AP001372.2 | 2.15282 | 0.04235 |
| 53 | ITGA1 | 2.50627 | 0.0004 |
| 54 | CSF1 | 3.26495 | 0.0031 |
| 55 | LINC01033 | inf | 0.0012 |
| 56 | CD83 | 2.10746 | 0.00005 |
| 57 | AC073135.3 | 2.17699 | 0.0215 |
| 58 | RPL9P7 | 2.12669 | 0.01975 |
| 59 | DIAPH3 | 2.57228 | 0.02155 |
| 60 | BHLHE22 | 2.07719 | 0.00005 |
| 61 | EPDR1 | 2.22225 | 0.03325 |
| 62 | EFNA1 | 2.30303 | 0.02205 |
| 63 | NFKB1 | 2.60625 | 0.0332 |
| 64 | SLC25A14 | 13.1153 | 0.04645 |
| 65 | FRMD4A | 2.40509 | 0.0097 |
| 66 | NFKB1 | 2.38521 | 0.0171 |
| 67 | MIR146A | inf | 0.00005 |
| 68 | PHLDB2 | 1.80605 | 0.00005 |
| 69 | DCX | 2.02 | 0.00025 |
| 70 | RP11-366M4.3 | 2.05705 | 0.012 |
| 71 | CNTFR | 3.90651 | 0.0476 |
| 72 | CRABP2 | 1.99188 | 0.03615 |
| 73 | MYEOV | 1.90743 | 0.00525 |
| 74 | DCX | 2.35224 | 0.0064 |
| 75 | RAB32 | 2.55056 | 0.03425 |
| 76 | SORL1 | 4.52615 | 0.0117 |
| 77 | AKAP12 | 1.73198 | 0.0001 |
| 78 | CFH | 1.95871 | 0.00335 |
| 79 | B4GALT1 | 1.80287 | 0.0001 |
| 80 | TMEM215 | 4.30075 | 0.03435 |
| 81 | NFE2L3 | 1.99397 | 0.0077 |
| 82 | MAGEC1 | 2.14764 | 0.0044 |
| 83 | SIX3 | 2.98316 | 0.01755 |
| 84 | MAP3K8 | 2.8109 | 0.0384 |
| 85 | TNFRSF4 | inf | 0.00095 |
| 86 | LRRC20 | 12.6786 | 0.04055 |
| 87 | HCG22 | 1.88899 | 0.03615 |
| 88 | CFL1 | inf | 0.04505 |
| 89 | MYLK | 1.77715 | 0.00025 |
| 90 | PITPNB | 1.76266 | 0.0455 |
| 91 | WWC3 | 2.01996 | 0.0005 |
| 92 | DDIT4 | 1.69051 | 0.005 |
| 93 | FBXO44 | 13.0416 | 0.0492 |
| 94 | MMP2 | 13.6455 | 0.0287 |
| 95 | TBX3 | 1.94141 | 0.0458 |
| 96 | APP | 1.47412 | 0.04865 |
| 97 | AKAP12 | 1.60956 | 0.0123 |
| 98 | RYR2 | 2.10743 | 0.00085 |
| 99 | MYADM | 13.2428 | 0.03975 |
| 100 | TMEM178B | 1.57051 | 0.0002 |
| 101 | FBLN1 | 1.45855 | 0.00195 |
| 102 | RP11-497H16.7 | inf | 0.02615 |
| 103 | IFNGR2 | 1.5083 | 0.02025 |
| 104 | JAK3 | 2.22437 | 0.0132 |
| 105 | WIPF3 | 2.23009 | 0.0214 |
| 106 | CA8 | inf | 0.00135 |
| 107 | TIFA | 1.76786 | 0.0241 |
| 108 | KCNB2 | inf | 0.00005 |
| 109 | HP1BP3 | 1.54847 | 0.03875 |
| 110 | KIAA1671 | 1.83896 | 0.04165 |
| 111 | JUN | 1.60125 | 0.00365 |
| 112 | FAM73A | 1.56624 | 0.0015 |
| 113 | TAP1 | 1.83736 | 0.0268 |
| 114 | LRRC37A3 | 13.2133 | 0.04305 |
| 115 | DNAJA1 | 1.51362 | 0.00005 |
| 116 | RAX | 1.78893 | 0.01275 |
| 117 | VARS2 | 12.4913 | 0.04875 |
| 118 | SPATA13 | 1.58447 | 0.0032 |
| 119 | ARNT2 | 2.88821 | 0.00445 |
| 120 | TMSB4X | 1.41226 | 0.0013 |
| 121 | SIX4 | 1.88489 | 0.0056 |
| 122 | ATP11A | 1.88978 | 0.0468 |
| 123 | MYPN | 2.17979 | 0.0263 |
| 124 | TNFRSF21 | 2.0263 | 0.03415 |
| 125 | GFRA2 | 1.75401 | 0.04285 |
| 126 | PPP1R14C | 2.53214 | 0.03175 |
| 127 | CPNE8 | 3.05924 | 0.0165 |
| 128 | MYO16 | 3.82674 | 0.02305 |
| 129 | NFKBIE | 1.55581 | 0.0405 |
| 130 | RP11-215G15.5 | 1.94825 | 0.0399 |
| 131 | AC022816.2 | inf | 0.0008 |
| 132 | HS6ST2 | 1.64436 | 0.0186 |
| 133 | HIPK2 | 1.46316 | 0.0004 |
| 134 | OAS3 | 1.38112 | 0.00075 |
| 135 | ZNF849P | 13.3616 | 0.03435 |
| 136 | HUNK | 3.06196 | 0.0166 |
| 137 | HMX2 | inf | 0.00015 |
| 138 | BHLHE40 | 3.63925 | 0.0389 |
| 139 | FOXO1 | 2.41048 | 0.0158 |
| 140 | CDK11B | 1.43283 | 0.0351 |
| 141 | LIMCH1 | 14.5934 | 0.02705 |
| 142 | MYCBP2 | 1.39805 | 0.01395 |
| 143 | CACHD1 | 1.25834 | 0.02355 |
| 144 | TRAF3 | 1.24306 | 0.00595 |
| 145 | UST | 1.66285 | 0.0495 |
| 146 | DCHS2 | 3.96101 | 0.00375 |
| 147 | HMGB1P5 | 1.19398 | 0.0242 |
| 148 | CNTNAP2 | 1.19903 | 0.0015 |
| 149 | SMU1 | 1.19915 | 0.04925 |
| 150 | ZFAND2B | 14.1545 | 0.047 |
| 151 | ETS2 | 1.2443 | 0.00495 |
| 152 | FRK | 2.65514 | 0.004 |
| 153 | PALB2 | 13.6014 | 0.0431 |
| 154 | MSRB3 | 14.3572 | 0.04095 |
| 155 | IFNAR1 | 1.32152 | 0.01075 |
| 156 | BICC1 | 2.54826 | 0.0277 |
| 157 | MAB21L3 | 1.45979 | 0.0439 |
| 158 | ADAMTS1 | 1.43536 | 0.01185 |
| 159 | DLX3 | 1.4537 | 0.0363 |
| 160 | SYT1 | 1.2991 | 0.0022 |
| 161 | EGFR | inf | 0.00005 |
| 162 | FAM214B | 14.8358 | 0.0231 |
| 163 | TLR7 | inf | 0.00005 |
| 164 | DSP | 1.96756 | 0.00775 |
| 165 | SOX2 | 1.2451 | 0.00425 |
| 166 | RGL1 | 1.28925 | 0.00495 |
| 167 | HLA-A | 1.18232 | 0.0244 |
| 168 | YPEL5 | 1.14621 | 0.01415 |
| 169 | MAGED4 | 11.0092 | 0.03885 |
| 170 | PARP14 | 1.32917 | 0.0149 |
| 171 | USP40 | 1.27902 | 0.0147 |
| 172 | ENPP4 | 1.43416 | 0.02845 |
| 173 | ALK | 1.54101 | 0.0233 |
| 174 | ZNF221 | 15.6167 | 0.0387 |
| 175 | SMAD9 | 1.26464 | 0.045 |
| 176 | SDC4 | 1.22582 | 0.0422 |
| 177 | CYYR1 | 1.11093 | 0.00345 |
| 178 | C2CD2 | 1.37119 | 0.02155 |
| 179 | VCP | 1.10828 | 0.0044 |
| 180 | LRCH1 | 1.23914 | 0.0156 |
| 181 | KIAA1549 | 1.82557 | 0.0162 |
| 182 | MGAT3 | 1.36512 | 0.02365 |
| 183 | DNAJC12 | 1.13165 | 0.0173 |
| 184 | ABCA5 | 15.3273 | 0.03835 |
| 185 | CSAG2 | inf | 0.02155 |
| 186 | AC079753.4 | inf | 0.0274 |
| 187 | RP1-104O17.3 | inf | 0.0224 |
| 188 | CRYGN | inf | 0.03965 |
| 189 | RP11-122C5.1 | inf | 0.022 |
| 190 | RP11-497H16.7 | inf | 0.03535 |
| 191 | LINC00871 | inf | 0.0186 |
| 192 | PLA2G4C | inf | 0.04625 |
| 193 | RP11-540O11.1 | inf | 0.03575 |
| 194 | RP11-757O6.4 | inf | 0.03575 |
| 195 | RP11-62H20.1 | inf | 0.03655 |
| 196 | CTD-2619J13.16 | inf | 0.03575 |
| 197 | RP11-425D10.10 | inf | 0.01495 |
| 198 | RP11-89C3.3 | inf | 0.03575 |
| 199 | SPINK2 | inf | 0.0306 |
| 200 | OR7E101P | inf | 0.04305 |
| 201 | RP11-599B13.3 | inf | 0.03575 |
| 202 | AC008746.12 | inf | 0.03575 |
| 203 | FCGBP | 1.74474 | 0.0358 |
| 204 | PCDHB14 | 13.0804 | 0.0361 |
| 205 | CHI3L1 | inf | 0.005 |
| 206 | RLN2 | inf | 0.0218 |
| 207 | RP11-269F21.3 | inf | 0.0279 |
| 208 | HSD17B14 | inf | 0.008 |
| 209 | RP1-302G2.5 | inf | 0.02115 |
| 210 | RP11-474I16.8 | inf | 0.0274 |
| 211 | AC137934.1 | inf | 0.01715 |
| 212 | RP11-345K20.2 | inf | 0.03575 |
| 213 | LINC00882 | inf | 0.01875 |
| 214 | RP11-70P17.1 | inf | 0.0274 |
| 215 | RP11-707G14.7 | inf | 0.03575 |
| 216 | C6orf58 | inf | 0.0012 |
| 217 | PKM | inf | 0.0446 |
| 218 | IL12RB2 | 14.2544 | 0.03085 |
| 219 | SULT1A3 | 10.8325 | 0.04695 |
| 220 | RP11-421N8.1 | inf | 0.0274 |
| 221 | MAPK11 | 14.9081 | 0.0331 |
| 222 | DAP3P2 | inf | 0.0274 |
| 223 | CCDC160 | inf | 0.0063 |
| 224 | TUBA1B | inf | 0.02905 |
| 225 | KLHL2P1 | inf | 0.0282 |
| 226 | RP11-575H3.1 | inf | 0.0274 |
| 227 | CTC-360P9.3 | inf | 0.02785 |
| 228 | FUT9 | 1.39428 | 0.04795 |
| 229 | MPDU1 | inf | 0.04885 |
| 230 | RSL24D1P6 | inf | 0.03575 |
| 231 | IGHV3-49 | inf | 0.022 |
| 232 | COL12A1 | 3.52109 | 0.02105 |
| 233 | FGFR1OP | 1.1284 | 0.0337 |
| 234 | EMB | inf | 0.00005 |
| 235 | ADAL | 13.2459 | 0.0395 |
| 236 | CLCN2 | inf | 0.0464 |
| 237 | HIVEP1 | 1.0804 | 0.03525 |
| 238 | RP11-524D16__A.3 | inf | 0.0139 |
| 239 | SLC25A13 | 11.76 | 0.04205 |
| 240 | PLCB4 | 15.2309 | 0.0305 |
| 241 | JAG1 | 1.88411 | 0.0421 |
| 242 | STC1 | 1.06379 | 0.04745 |
| 243 | ZIC5 | 1.05444 | 0.03225 |
| 244 | OR2I1P | inf | 0.0022 |
| 245 | FAM131A | inf | 0.04675 |
| 246 | IRS2 | 1.01041 | 0.00945 |
| 247 | AHDC1 | 1.11337 | 0.0339 |
| 248 | TBX3 | 1.06529 | 0.0467 |
| 249 | RP11-51F16.9 | inf | 0.022 |
| 250 | FBN2 | 1.03043 | 0.0146 |
| 251 | AC016907.3 | inf | 0.02205 |
| 252 | NEDD9 | 2.55427 | 0.035 |
| 253 | NOL6 | 1.04924 | 0.0069 |
| 254 | NCBP2 | 1.02918 | 0.025 |
| 255 | SCNN1G | 1.06806 | 0.0135 |
| 256 | RP11-316P17.2 | inf | 0.0014 |
| 257 | APP | 1.03186 | 0.01625 |
| 258 | CBFA2T2 | 13.4467 | 0.04605 |
| 259 | HSPA12A | 2.97913 | 0.04415 |
| 260 | CHMP5 | 1.01098 | 0.01685 |
| 261 | KDM8 | 12.2933 | 0.04195 |
| 262 | TRIM69 | 1.06048 | 0.03085 |
| 263 | TOLLIP | 1.11623 | 0.04475 |
| 264 | DMXL1 | 13.9382 | 0.023 |
| 265 | MARCKS | 1.13376 | 0.0177 |
| 266 | ZNF462 | 1.95268 | 0.022 |
| 267 | KCNE4 | 1.13057 | 0.0292 |
| 268 | SLC25A30 | 1.09825 | 0.00965 |
| 269 | ACO1 | 1.11142 | 0.02105 |
| 270 | COTL1 | 1.08236 | 0.01895 |
| 271 | PIM3 | 1.08116 | 0.01725 |
| 272 | CAV1 | 1.04173 | 0.0098 |
| 273 | HLA-DRA | inf | 0.0006 |
| 274 | TMEM2 | 1.4771 | 0.0377 |
| 275 | RNF38 | 1.21849 | 0.0408 |
| 276 | WASH2P | 13.8817 | 0.04605 |
| 277 | KB-1027C11.4 | inf | 0.01495 |
| **Down-regulated** |  |  |  |
| 278 | GSPT1 | -1.3355 | 0.01325 |
| 279 | SLC17A7 | -1.88987 | 0.0424 |
| 280 | ALDH1L2 | -2.46246 | 0.0166 |
| 281 | CDH3 | -2.58915 | 0.02995 |
| 282 | SLC29A2 | -1.33501 | 0.0197 |
| 283 | INF2 | -12.9896 | 0.0465 |
| 284 | MYO1D | -1.46038 | 0.0117 |
| 285 | C3AR1 | -2.36301 | 0.04715 |
| 286 | LIG4 | -13.0462 | 0.0396 |
| 287 | CPE | -1.31439 | 0.00835 |
| 288 | CACNA1H | -1.71854 | 0.00865 |
| 289 | PDE8A | -11.9206 | 0.0493 |
| 290 | RPS2 | #NAME? | 0.03845 |
| 291 | FLYWCH2 | -1.3939 | 0.0368 |
| 292 | EMR1 | -12.3508 | 0.03445 |
| 293 | ZNF502 | -13.2581 | 0.0266 |
| 294 | PCSK9 | -2.78154 | 0.0444 |
| 295 | TAB3 | -1.62693 | 0.04075 |
| 296 | PARD3 | -13.2421 | 0.0498 |
| 297 | GNG4 | -1.81715 | 0.03055 |
| 298 | HAPLN1 | -3.70144 | 0.0271 |
| 299 | TKT | -1.42367 | 0.0405 |
| 300 | PAGE5 | #NAME? | 0.00075 |
| 301 | CERCAM | -1.65295 | 0.0263 |
| 302 | F8A3 | -2.67324 | 0.0304 |
| 303 | CLVS2 | -1.70375 | 0.0014 |
| 304 | ORAOV1 | #NAME? | 0.0372 |
| 305 | PYGL | -1.53892 | 0.01355 |
| 306 | QPRT | -1.37258 | 0.0021 |
| 307 | ENPEP | -1.78492 | 0.0064 |
| 308 | TCEA3 | -1.53826 | 0.0043 |
| 309 | C14orf93 | -14.7093 | 0.02585 |
| 310 | HPDL | -1.68503 | 0.018 |
| 311 | KRT18 | -1.50271 | 0.0042 |
| 312 | ZNF367 | -1.62266 | 0.0043 |
| 313 | PRKCB | -1.51297 | 0.00015 |
| 314 | TRMT2B | -12.7783 | 0.0324 |
| 315 | CDCA7 | -1.49672 | 0.00835 |
| 316 | DFNB31 | -14.8535 | 0.02665 |
| 317 | RALGPS2 | -1.60754 | 0.00185 |
| 318 | KIAA2022 | -4.00685 | 0.0038 |
| 319 | SELENBP1 | -1.60808 | 0.0422 |
| 320 | RWDD4 | -1.71606 | 0.0499 |
| 321 | GPT2 | -1.84065 | 0.01955 |
| 322 | RP11-478C6.5 | #NAME? | 0.01295 |
| 323 | GPR124 | -1.66977 | 0.00215 |
| 324 | LGR4 | -2.22283 | 0.0381 |
| 325 | LTBP4 | -2.18102 | 0.04585 |
| 326 | IDH1 | -1.57924 | 0.00075 |
| 327 | GSPT1 | -1.10365 | 0.0498 |
| 328 | RHOB | -1.19347 | 0.0374 |
| 329 | PHGDH | -1.08365 | 0.00355 |
| 330 | VIM | -1.09231 | 0.0063 |
| 331 | MAP3K5 | -2.23763 | 0.0205 |
| 332 | NPY1R | -1.19959 | 0.02275 |
| 333 | DHCR24 | -1.11767 | 0.00265 |
| 334 | SLC38A1 | -1.16789 | 0.0018 |
| 335 | FAM175A | -1.18163 | 0.04485 |
| 336 | ZIC3 | -2.62134 | 0.0268 |
| 337 | GATA2 | -13.3004 | 0.0398 |
| 338 | FHOD1 | -1.38112 | 0.0285 |
| 339 | TEX15 | -1.34189 | 0.0049 |
| 340 | MAP3K6 | -15.4931 | 0.04855 |
| 341 | FAM129B | -1.28407 | 0.0109 |
| 342 | BAZ1A | -1.2319 | 0.05 |
| 343 | EXOC6 | -1.35615 | 0.05 |
| 344 | NES | -1.61266 | 0.024 |
| 345 | MYO5A | -2.19131 | 0.0359 |
| 346 | ADRA1D | -1.2452 | 0.0166 |
| 347 | CES1 | -1.19866 | 0.03345 |
| 348 | SCD | -1.18368 | 0.001 |
| 349 | TXNIP | -1.20434 | 0.00265 |
| 350 | MAGEA12 | #NAME? | 0.0005 |
| 351 | MAPK8IP1 | -1.94185 | 0.0404 |
| 352 | CAPN6 | -1.25689 | 0.00335 |
| 353 | AL807752.1 | #NAME? | 0.01635 |
| 354 | TRAK1 | -1.14978 | 0.0377 |
| 355 | FAM210B | -1.11191 | 0.04705 |
| 356 | ABCB10 | -1.0872 | 0.02235 |
| 357 | TDRD7 | -15.5208 | 0.04715 |
| 358 | ARC | -2.66608 | 0.02665 |
| 359 | CNTN6 | -15.8089 | 0.0453 |
| 360 | ARHGEF17 | -1.18859 | 0.01335 |
| 361 | ZCCHC24 | -1.21334 | 0.0264 |
| 362 | ZNF845 | -12.9742 | 0.0354 |
| 363 | BTN3A3 | -12.4566 | 0.04045 |
| 364 | PRDM10 | -12.8068 | 0.0461 |
| 365 | LPXN | -11.6507 | 0.04985 |
| 366 | ANXA1 | -1.05951 | 0.02325 |
| 367 | TGFBR1 | -1.29596 | 0.04855 |
| 368 | VN1R83P | #NAME? | 0.01615 |
| 369 | TSPAN13 | -1.06307 | 0.0216 |
| 370 | CYP4F22 | -1.12877 | 0.0411 |
| 371 | NHS | -2.50311 | 0.01165 |
| 372 | ESYT1 | -1.08688 | 0.0169 |
| 373 | WNK2 | -14.6132 | 0.03655 |
| 374 | PLCB1 | -14.8995 | 0.0468 |
| 375 | RP11-196G18.22 | -1.28728 | 0.03295 |
| 376 | ASIC2 | -1.22505 | 0.04765 |
| 377 | IKBKB | -11.9812 | 0.0437 |
| 378 | KIAA1958 | -1.3329 | 0.0352 |
| 379 | ADCY9 | -1.39082 | 0.01655 |
| 380 | UNC5B | -2.25836 | 0.0046 |
| 381 | CLEC11A | -1.69489 | 0.0018 |
| 382 | GBP6 | -4.4252 | 0.04765 |
| 383 | CTD-2311M21.2 | #NAME? | 0.00005 |
| 384 | SORBS3 | -1.95836 | 0.0445 |
| 385 | METTL7A | -2.11149 | 0.01475 |
| 386 | PRKD2 | -14.0659 | 0.0294 |
| 387 | FDFT1 | -1.65146 | 0.01395 |
| 388 | CYP24A1 | -13.1769 | 0.03995 |
| 389 | GNG4 | -1.75631 | 0.0224 |
| 390 | DNAJC15 | -3.69039 | 0.04125 |
| 391 | HSPB8 | -2.86805 | 0.0202 |
| 392 | HMGA2 | -1.83164 | 0.00025 |
| 393 | GPD2 | -2.27815 | 0.0029 |
| 394 | TMEM117 | -2.23345 | 0.02535 |
| 395 | INA | -2.15493 | 0.01585 |
| 396 | ZNF189 | -13.6071 | 0.0385 |
| 397 | CCSAP | -2.10779 | 0.018 |
| 398 | CD248 | -1.74515 | 0.00065 |
| 399 | PSMD2 | #NAME? | 0.03495 |
| 400 | KCNA2 | -3.30428 | 0.0159 |
| 401 | ASS1 | -2.3199 | 0.0414 |
| 402 | KCNQ2 | #NAME? | 0.00005 |
| 403 | EXOC3L1 | -3.26619 | 0.03275 |
| 404 | ZSCAN9 | -14.589 | 0.04365 |
| 405 | MAGEA3 | #NAME? | 0.00005 |
| 406 | PARP1 | -1.73791 | 0.00005 |
| 407 | PRIMPOL | -12.7874 | 0.0456 |
| 408 | AR | -2.52573 | 0.00005 |
| 409 | FAM181B | -2.54716 | 0.01065 |
| 410 | BRINP1 | -2.89449 | 0.01195 |
| 411 | CUX2 | -3.33463 | 0.0033 |
| 412 | S100A11 | -2.07932 | 0.0013 |
| 413 | DPPA4 | #NAME? | 0.00005 |
| 414 | RPL21P75 | -2.01787 | 0.0165 |
| 415 | GRIK3 | -4.63304 | 0.034 |
| 416 | SRSF5 | -1.96803 | 0.0276 |
| 417 | MYH14 | -5.1595 | 0.0361 |
| 418 | CBS | -2.09295 | 0.0009 |
| 419 | SRCAP | #NAME? | 0.0418 |
| 420 | RP11-157B13.6 | #NAME? | 0.0254 |
| 421 | VN1R110P | #NAME? | 0.01295 |
| 422 | NOL5BP | #NAME? | 0.0449 |
| 423 | RP4-673D20.3 | #NAME? | 0.0013 |
| 424 | CTC-548K16.1 | #NAME? | 0.01515 |
| 425 | HIST1H2BE | #NAME? | 0.0254 |
| 426 | SOX17 | #NAME? | 0.00055 |
| 427 | TUFT1 | -14.9753 | 0.03755 |
| 428 | ZNF185 | -12.505 | 0.0384 |
| 429 | TEX26 | #NAME? | 0.011 |
| 430 | LAMB3 | -13.5056 | 0.03925 |
| 431 | KIAA0040 | -2.28558 | 0.04725 |
| 432 | EIF4G1 | #NAME? | 0.04035 |
| 433 | RIN3 | -13.3067 | 0.0393 |
| 434 | ZNF23 | -12.5922 | 0.0449 |
| 435 | AC093673.5 | #NAME? | 0.01295 |
| 436 | RP11-187C18.3 | #NAME? | 0.01615 |
| 437 | CNTN4 | -12.0305 | 0.05 |
| 438 | RP11-734I18.1 | #NAME? | 0.0118 |
| 439 | SGK3 | -11.3419 | 0.049 |
| 440 | RP4-595K12.2 | #NAME? | 0.0122 |
| 441 | HIST2H2BD | #NAME? | 0.01635 |
| 442 | COX20 | #NAME? | 0.0439 |
| 443 | FAM214B | -13.3758 | 0.03905 |
| 444 | SETBP1 | -1.56771 | 0.02815 |
| 445 | LIN7A | -1.01199 | 0.0306 |
| 446 | NYNRIN | -1.02122 | 0.01585 |
| 447 | AMBN | -13.4053 | 0.0416 |
| 448 | GAPDH | #NAME? | 0.0261 |
| 449 | RP11-10C24.1 | #NAME? | 0.0449 |
| 450 | AC093510.1 | #NAME? | 0.03285 |
| 451 | PSMA2P1 | #NAME? | 0.03285 |
| 452 | RP11-166P13.3 | #NAME? | 0.04705 |
| 453 | CDA | #NAME? | 0.0154 |
| 454 | CTD-2085J24.4 | #NAME? | 0.0449 |
| 455 | RP11-697E2.9 | #NAME? | 0.0197 |
| 456 | RP11-553A10.1 | #NAME? | 0.0206 |
| 457 | HIST1H3B | #NAME? | 0.0449 |
| 458 | CTC-296K1.4 | #NAME? | 0.03285 |
| 459 | RP4-593C16.3 | #NAME? | 0.03285 |
| 460 | RP11-342K6.4 | #NAME? | 0.01635 |
| 461 | TCEAL5 | #NAME? | 0.01295 |
| 462 | FAM183A | #NAME? | 0.03885 |
| 463 | LINC00334 | #NAME? | 0.0336 |
| 464 | SERP2 | #NAME? | 0.01515 |
| 465 | RP11-434P11.2 | #NAME? | 0.03285 |
| 466 | RP1-30G7.2 | #NAME? | 0.0254 |
| 467 | VPREB3 | #NAME? | 0.04215 |
| 468 | DVL3 | #NAME? | 0.0441 |
| 469 | RP1-266L20.2 | #NAME? | 0.0424 |
| 470 | RP11-435O5.2 | #NAME? | 0.00985 |
| 471 | C6orf132 | -2.36954 | 0.0428 |
| 472 | RP11-109L13.1 | #NAME? | 0.01635 |
| 473 | HIST2H3D | #NAME? | 0.0449 |
| 474 | CTD-2550O8.7 | #NAME? | 0.0449 |
| 475 | AC092415.1 | #NAME? | 0.0334 |
| 476 | CTC-296K1.3 | #NAME? | 0.0449 |
| 477 | RP11-277P12.9 | #NAME? | 0.0449 |
| 478 | RP11-293M10.5 | #NAME? | 0.0336 |
| 479 | RP11-1250I15.3 | #NAME? | 0.0449 |
| 480 | CTD-2537I9.5 | #NAME? | 0.0449 |
| 481 | MSH6 | #NAME? | 0.0347 |
| 482 | MT1E | #NAME? | 0.00005 |
| 483 | OLFM1 | -3.82635 | 0.03395 |
| 484 | STARD13 | -2.67323 | 0.0029 |
| 485 | BTBD17 | -3.33131 | 0.0075 |
| 486 | PRRC2C | #NAME? | 0.01775 |
| 487 | SRRM1 | #NAME? | 0.03935 |
| 488 | COQ10A | -13.8839 | 0.02295 |
| 489 | MAGEA6 | #NAME? | 0.00005 |
| 490 | CTD-2311M21.2 | #NAME? | 0.0003 |
| 491 | UBE2Q2P6 | -4.38124 | 0.0462 |
| 492 | MT1G | -4.43589 | 0.0254 |
