# Supplementary material for: Polymerase independent repression of FoxO1 transcription by sequence-specific PARP1 binding to FoxO1 promoter
Source: Cell Death Dis. 2020 Jan 28;11(1):71. doi: 10.1038/s41419-020-2265-y (PMC6987093; doi:10.1038/s41419-020-2265-y)
Supplement: Supplementary file 7 — Supplementary Table S2 [file 41419_2020_2265_MOESM7_ESM.doc]

**Supplementary Table S2.**

| **No.** | **Genes** | **Log_2_ Fold Change** | ***p*-value** |
| --- | --- | --- | --- |
| **Up-regulated** |  |  |  |
| 1 | TNFAIP3 | 5.80766 | 0.00055 |
| 2 | NF-κB2 | 3.70595 | 0.01115 |
| 3 | NF-κB1 | 3.19954 | 0.00275 |
| 4 | NF-κBIA | 2.72184 | 0.00005 |
| 5 | NF-κB2 | 2.68852 | 0.0007 |
| 6 | NF-κB1 | 2.60625 | 0.0332 |
| 7 | FOXO1 | 2.41048 | 0.0158 |
| 8 | NF-κB1 | 2.38521 | 0.0171 |
| 9 | JUN | 1.60125 | 0.00365 |
| 10 | TRAF3 | 1.24306 | 0.00595 |
| **Down-regulated** |  |  |  |
| 11 | IκBKB | -11.9812 | 0.0437 |
| 12 | AR | -2.52573 | 0.00005 |
| 13 | PRKCB | -1.51297 | 0.00015 |
