# Supplementary material for: Polymerase independent repression of FoxO1 transcription by sequence-specific PARP1 binding to FoxO1 promoter
Source: Cell Death Dis. 2020 Jan 28;11(1):71. doi: 10.1038/s41419-020-2265-y (PMC6987093; doi:10.1038/s41419-020-2265-y)
Supplement: Supplementary file 8 — Supplementary Table S3 [file 41419_2020_2265_MOESM8_ESM.doc]

**Supplementary Table S3.**

| **Location** | **Nucleotide sequence** | **Location** |
| --- | --- | --- |
| -1 | GGCAGCGGCTGCTGCGACTACCAGGCCGCCCGACTTACGGGATCTGCCGC | -50 |
| -51 | CGCCCCCCGCCCGCGGCGGCGCGCGCGCCGGCCCGCCCCTGACCGACAGC | -100 |
| -101 | CCGCGCGGCCAATGGGCATGCGGCACCGCCGCCCGGGCAGCCAGTGGGCG | -150 |
| -151 | CCGGGCTGGGTGGGGCCCGGTTTTCCACGGGGAGGCGGCGGTGGGCTGGT | -200 |
| -201 | GGGGGGTAGTGGGGTGTTTTTCTCTTTCACACACTCACCTCCTTTTTTTT | -250 |
| -251 | TTTTTGGATCTCTATTATTTTCTGGTAATTCTCGAGTGTTTCTGTGATTC | -300 |
| -301 | TCTCGCCTTCTCAGTGTTTTGATTGCTAGGAAGCAAACCAGCGTGGAGGC | -350 |
| -351 | GCCGGCGACACTTTGTTTACTACGGAGCAGCAGAGCCGAGTACTCGGGAA | -400 |
| -401 | GCCCGGGTGGGAGGAGGCGCTCGCTGCTCCCTGACCTCCGCTGCGGGCCG | -450 |
| -451 | AGCCCGGCGGGCTGGCAGGGCAGGGGGCCGAGGGCCGGGGGCGCGGGGTG | -500 |
| -501 | GGCGGGCGGAGGCGGCCGCGAGGAATTCTACTCAATCGCTCCCTCCTGGC | -550 |
| -551 | TCCACCCACGATGTCTTTGCTGAACGACGTGGGGAATCGGTGGGTTTTGT | -600 |
| -601 | TTTGGTTTAATGTTTTCTTTCGCTGCGATCTGTCAAGTCCTCCGGCCCCC | -650 |
| -651 | TCGCGAGCGGCACACGCCCCCCACCCCCGGCGCCGCGGCTCCTGCAGTCG | -700 |
| -701 | AGTCCGCGCCGAGGGACCCTTCTCCGTGCCCACCGGTCCGCACCCCCGGG | -750 |
| -751 | CTCAGCCTGTGCCATTCGGTCTAGCCAGAGGCGCGTCACCCAGCCGGCAG | -800 |
| -801 | CCCCCAGCCGGAT**TCACTGTATTCTT**GACCTTTTTTAAAATGCTAGAAAT | -850 |
| -851 | GAGGAACAGGGGCTCCGGCGCGGGGAGGAGATTATCTGGCCTCCCTGAAC | -900 |
| -901 | ACACGAATCCAAAGGCACCCGCGACCGCATGCCCTCCGTGGGAACCTCTT | -950 |
| -951 | CCCCTGGGCGGGAATCTCCCGCTCCGTCCACTAAGTCCAGGGCCGGCAGC | -1000 |
| -1001 | GAAGGGCTCAGGAGCGCAGTTAGAGAGAACCAGGTCATTTTATTACTTAT | -1050 |
| -1051 | CCCTAATTAAATTTAGCTGTTTGTTACTTCTTTTTTATTTGCAACGGAAG | -1100 |
| -1101 | TGATCGGGTGTTAAGTAAAAACGCAAGGGGGGCGGGAGATAGGACCAAAG | -1150 |
| -1151 | CCTTGGGGCGCAGCCTGCCCCACCCCCAAATTCCTTGAGGCTACTTGCTG | -1200 |
| -1201 | TGTGTGTCAGTCAACCCCCCACCCACACCCCACCTTTCCTCATCTGGGCT | -1250 |
| -1251 | GCAGGTCCAGGACATCCTGTCAAGGATCAGTGGGGCCAAAGGTGGGAAGA | -1300 |
| -1301 | TAATGGCCCTTGTGTCTCAAGCAGAAGAAACAGTTTTCCAGTTTTGTTTT | -1350 |
| -1351 | GTTATGCTTTTGCTTTTTTAGGAAGAATATGGAAGGCATTTCCTTTTAGA | -1400 |
| -1401 | AAGATGGGACCACTTAGGATGGAAGATGGACAGTAGGGTTTTAAATATCT | -1450 |
| -1451 | TGTCCAAAAGGACAGCTTCCAACTCTTGTTTTTTAATCCCTTTCTTTCTT | -1500 |
| -1501 | ACAAGTAATTCTGCAATGGAAAAAGTGGCATCTAGAGAAAATTAGTCAGT | -1550 |
| -1551 | TGGACAAGAGGTTGTTGAAAAAATAAAAGAGAAAAGAGGGGAAAAAAGGA | -1600 |
| -1601 | AAAATTAGGCCTCTTACAAAAGTTAAAAAGATTGAGAGAGATTATCTTTC | -1650 |
| -1651 | TTCTTTTATATTCTCACTATCGTCTCTCTAGTCCGGGCTCCTGTTTCTTC | -1700 |
| -1701 | ATAATCTATTTATCACAACAGCTTCTTGCTGATACTAGGCATGGGTGGGG | -1750 |
| -1751 | TGGGGAGCGTGAGCCGGATGGATTGGGGACGGGCGGGGACTGGTTCCCAC | -1800 |
| -1801 | CCTGG**TCTTGTGGTCTCTTCACGTTTAC**TATCAAATTTGGACAGTCTGGG | -1850 |
| -1851 | CCTCCTCTGACAGCCGCTAGGGGACCTCAGTAACTCAGATGATGAGCTCA | -1900 |
| -1901 | AAGGCAGGCCCCCAAAGTACAGCTGTTGCTTCCCCCCACCTTTCCAATTA | -1950 |
| -1951 | CACTCTGCAGAAGCAGCCCCCGATCCAGACAGCCGCACCCTAAAAATAGG | -2000 |
