# Supplementary material for: Polymerase independent repression of FoxO1 transcription by sequence-specific PARP1 binding to FoxO1 promoter
Source: Cell Death Dis. 2020 Jan 28;11(1):71. doi: 10.1038/s41419-020-2265-y (PMC6987093; doi:10.1038/s41419-020-2265-y)
Supplement: Supplementary file 9 — Supplementary Table S4 [file 41419_2020_2265_MOESM9_ESM.doc]

**Supplementary Table S4.**

| **Primer name** | **Primer sequences** |
| --- | --- |
| **Primers for real-time PCR:** | |
| *FoxO1* sense: | 5'-GTCAAGAGCGTGCCCTACTTC-3' |
| *FoxO1* antisense: | 5'-CCATGGATGCAGCTCTTCTC-3' |
| *TNFAIP3* sense: | 5'-AGGTTCCAATTTCGCCCCTT-3' |
| *TNFAIP3* antisense: | 5'-GATTTCAGGCCCACTGTCCT-3' |
| *NF-κBIA* (*IκBα*) sense: | 5'-AAGTGATCCGCCAGGTGAAG-3' |
| *NF-κBIA* (*IκBα*) antisense: | 5'-CTGCTCACAGGCAAGGTGTA-3' |
| *NF-κB1* sense: | 5'-GTTTGTCCAGCTTCGGAGGA-3' |
| *NF-κB1* antisense: | 5'-GACCTGTACTTCCAGTGCCC-3' |
| *p21* sense: | 5'-CAGCAGAGGAAGACCATGTG-3' |
| *p21* antisense | 5'-GAGGCACAAGGGTACAAGACA-3' |
| *PUMA* sense: | 5'-ACGACCTCAACGCACAGTACG-3' |
| *PUMA* antisense: | 5'-GTAAGGGCAGG AGTCCCATGATG-3' |
| *Bim* sense: | 5'-TAAGTTCTGAGTGTGACCGAGA-3' |
| *Bim* antisense: | 5'-GCTCTGTCTGTAGGGAGGTAGG-3' |
| *β-Actin* sense: | 5'-CTGGAACGGTGAAGGTGACA-3' |
| *β-Actin* antisense | 5'-AAGGGACTTCCTGTAACAATGCA -3' |
| **Primers used for ChIP in the *FoxO1* promoter:** | |
| *Primer L* sense: | 5'-AGCCTGTGCCATTCGGTCTA-3' |
| *Primer L* antisense: | 5'-GGGTGCCTTTGGATTCGTGT-3' |
| *Primer M* sense: | 5'-TGGGACCACTTAGGATGGAAG-3' |
| *Primer M* antisense: | 5'-ACAACCTCTTGTCCAACTGACT-3' |
| *Primer R* sense: | 5'-ACCCTGGTCTTGTGGTCTCT-3' |
| *Primer R* antisense: | 5'-AACAGCTGTACTTTGGGGGC-3' |
| **DNA fragments used for FREP** | |
| *FoxO1-L-B*-biotin | /5biosg/AATGATACGGCGACCACCGAGGATCCTCACTGTATTCTTGAATTCTCGTATGCCGTCTTCGCTTG |
| *FoxO1-L-B* | AATGATACGGCGACCACCGAGGATCCTCACTGTATTCTTGAATTCTCGTATGCCGTCTTCTGCTTG |
| *FoxO1-R-B*-biotin | /5biosg/AATGATACGGCGACCACCGAGGATCCTCTTGTGGTCTCTTCACGTTTACGAATTCTCGTATGCCGTCTTCTGCTTG |
| *FoxO1-R-B* | AATGATACGGCGACCACCGAGGATCCTCTTGTGGTCTCTTCACGTTTACGAATTCTCGTATGCCGTCTTCTGCTTG |
| Non-specific DNA sequence, *NS*-biotin | /5biosg/AATGATACGGCGACCACCGAGGATCCAGGGCTGTAGATTCCGGCCTGAAGCCTGGGAATTCTCGTATGCCGTCTTCTGCTTG |
