# Supplementary material for: Polymerase independent repression of FoxO1 transcription by sequence-specific PARP1 binding to FoxO1 promoter
Source: Cell Death Dis. 2020 Jan 28;11(1):71. doi: 10.1038/s41419-020-2265-y (PMC6987093; doi:10.1038/s41419-020-2265-y)
Supplement: Supplementary file 10 — Declaration of contribution to article [file 41419_2020_2265_MOESM10_ESM.pdf]

DECLARATION OF CONTRIBUTIONS TO ARTICLE

ADMC

Manuscript Number:	Journal Name:	
CDDIS-19-3136R	*Cell Death & Disease*	(the 'Journal')

Proposed Title of the Contribution:

Polymerase independent repression of FoxO1 transcription by sequence-specific PARP1 binding to FoxO1 promoter (the 'Contribution)

Author(s):

Yu-Nan Tian, Hua-Dong Chen, Chang-Qing Tian, Ying-Qing Wang, Ze-Hong Miao (the 'Authors')

For all *CDDis* articles, each person named as an author in the published version must be able to show he or she has contributed substantially to the article.

Authorship credit should be based on 1) substantial contributions to conception and design, acquisition of data, or analysis and interpretation of data; 2) drafting the article or revising it critically for important intellectual content; and 3) final approval of the version to be published. Authors should meet conditions 1, 2 and 3.

Any person who cannot be shown to have made a substantial contribution to the article cannot be listed as an author in the final version. The name of any person who is deemed to have made a minor contribution can, however, appear in the Acknowledgments section of the article.

Please complete the table below to indicate the contributions of all named authors to the manuscript.

Author Full Name:	Specification of Contribution to the Manuscript:
Yu-Nan Tian	designed the study,carried out experiments, interpreted the results, wrote the manuscript, and finally approved the version to be published.
Hua-Dong Chen	designed the study, carried out experiments, analyzed and interpreted the data, and finally approved the version to be published.
Chang-Qing Tian	carried out experiments, analyzed and interpreted the data, and finally approved the version to be published.
Ying-Qing Wang	designed the study,interpreted the results,wrote the manuscript,and finally approved the version to be published.
Ze-Hong Miao	designed the study,interpreted the results,wrote the manuscript,and finally approved the version to be published.

Please complete the table below to indicate the contributions of all named authors to the figures.

Figure 1:

A.Stable knockout of PARP1 cells were conducted by Hua-Dong Chen.
A,B,C,D,E,F,G were acquired by Yu-Nan Tian.

Figure 2:

A.Stable knockout of PARP1 with the CRISPR/Cas9 technique was conducted by Hua-Dong Chen.
D.Plasmid construction was conducted by Hua-Dong Chen.
A,B,C,D data were acquired by Yu-Nan Tian.

Figure 3:

C.ChIP data was acquired by Yu-Nan Tian with the help of Chang-Qing Tian.
A,B,C,D data were acquired by Yu-Nan Tian.

Figure 4:

A,B,C,D,E data were acquired by Yu-Nan Tian.

Figure 5:

B,C. Generation of cells expressing PARP1 mutants was conducted by Hua-Dong Chen.
E.Plasmid construction was conducted by Hua-Dong Chen.
A,B,C,D,E,F, G, H, I were acquired by Yu-Nan Tian.

Figure 6:

Signed for and on behalf of the Author(s):	Print Name:	Date:
Ze-Hong Miao	Ze-Hong Miao	Jan. 2, 2020.
